# Identification of novel candidate genes for regulating oil composition in soybean seeds under environmental stresses

**DOI:** 10.3389/fpls.2025.1572319

**Published:** 2025-04-17

**Authors:** Patrick Bewick, Peter Forstner, Bo Zhang, Eva Collakova

**Affiliations:** ^1^ School of Plant and Environmental Sciences, Virginia Polytechnic Institute and State University, Blacksburg, VA, United States; ^2^ Translational Plant Science Center, Virginia Polytechnic Institute and State University, Blacksburg, VA, United States; ^3^ Fralin Life Science Institute, Virginia Polytechnic Institute and State University, Blacksburg, VA, United States

**Keywords:** abiotic and biotic stresses, fatty acids, mGWAS, oil composition, phytohormone signaling, SNPs, soybean seeds

## Abstract

**Introduction:**

A key objective of soybean breeding programs is to enhance nutritional quality for human and animal consumption, with improved fatty acid (FA) composition for health benefits, and expand soybean use for industrial applications.

**Methods:**

We conducted a metabolite genome-wide association study (mGWAS) to identify genomic regions associated with changes in FA composition and FA ratios in soybean seeds influenced by environmental factors. This mGWAS utilized 218 soybean plant introductions (PIs) grown in two field locations in Virginia over two years.

**Results:**

The mGWAS revealed that 20 SNPs were significantly associated with 21 FA ratios, while additional suggestive SNPs were found for 36 FA ratios, highlighting potential quantitative trait loci linked to FA composition.

**Discussion:**

Many of these SNPs are located near or within the genes related to phytohormone-mediated biotic and abiotic stress responses, suggesting the involvement of environmental factors in modulating FA composition in soybean seeds. Our findings provide novel insights into the genetic and environmental factors influencing FA composition in oilseeds. This research also lays the foundation for developing stable markers to develop soybean cultivars with tailored FA profiles for different practical applications under variable growth conditions.

## 1 Introduction

Since its domestication in East Asia 6,000-9,000 years ago, genetic diversity in soybeans has decreased by approximately 50%, with an 81% loss of rare alleles (Sedivy et al., 2017). A similar reduction in nucleotide diversity was observed for genes involved with fatty acid (FA) metabolism, which can be attributed to desirable trait selection and retention, genetic bottlenecks, and/or genetic drift (Derbyshire et al., 2023). Now, soybean oil typically contains approximately 10% palmitic acid (PA), 20% oleic acid (OA), 55% linoleic acid (LA), and 10% alpha-linolenic acid (ALA) representing the major FAs (Song et al., 2023). With its low saturated FAs (SFAs) content and elevated levels of monounsaturated (MUFAs) and polyunsaturated FAs (PUFAs), soybean oil is often considered as health promoting in humans and animals (Martins et al., 2023; Natnan et al., 2023; Tutunchi et al., 2020). Due to the health-promoting properties of OA and because less saturation in FAs is desirable for oil stability, breeding programs have focused on creating soybean cultivars with seed oil enriched in OA (Baer et al., 2021; Huth et al., 2015; Sakurai et al., 2003), contributing to the loss of genetic diversity in soybean cultivars.

However, high-OA soybean oil may not be suitable for other applications as it typically has lower levels of LA and ALA. For example, these two FAs are precursors for high-value ω-3 FAs [eicosapentaenoic acid (EPA) and docosahexaenoic acid (DHA)] that are essential for human and animal diets and health (Banaszak et al., 2024; Li et al., 2021; Swanson et al., 2012). The major EPA and DHA dietary sources are algae and fish oil. While fish acquire these FAs predominantly from their diets, some algae possess several FA desaturases and elongases capable of EPA and DHA biosynthesis from LA and ALA (Mühlroth et al., 2013). Plants, on the other hand, do not produce detectable levels of these very-long chain PUFAs, but they can be engineered to biosynthesize them (Petrie et al., 2012; Ruiz-Lopez et al., 2015; Usher et al., 2017; Walsh et al., 2016). Identifying soybean PIs with high LA and ALA content or specific FA ratios provides the basis for future soybean breeding or engineering for diverse specific practical applications.

Although soybean cultivars have been selected based on specific FA content, FA composition of soybean oil can vary in response to environment. Soybean seed FA composition is influenced by heat and drought stress as well as insect feeding (Bellaloui et al., 2015; Kanobe et al., 2015; Quoc Thien et al., 2016; Sehgal et al., 2018). Interestingly, these types of abiotic and biotic stresses have synergistic effects on FA composition in membrane lipids and seed oil as they reduce PUFAs like LA and ALA while increasing PA and OA not only in soybean (Schmidt et al., 2015), but also in other plant species (Mousavi et al., 2022; Wang et al., 2022a). These changes in FA composition are essential for maintaining membrane fluidity and function under drought and heat (Henschel et al., 2024; Higashi and Saito, 2019; Sharma et al., 2023). In the case of insect feeding, they are a result of an insect defense mechanism to block jasmonic acid (JA) signaling by limiting the availability of ALA for JA synthesis (Kanobe et al., 2015; Thompson and Goggin, 2006). Considering that JA is derived from ALA (Sharma et al., 2023), there is also a direct metabolic relationship between JA and FA composition in addition to regulatory interactions. JA and salicylic acid (SA) are important components of molecular defense mechanisms against herbivory (Mostafa et al., 2022). Application of JA and SA to soybean leaves resulted in changes in FA composition (Ghassemi-Golezani and Farhangi-Abriz, 2018), suggesting their involvement in an overall regulation of FA composition during herbivory and plant diseases.

In addition to JA, other phytohormones are also involved in modulating FA composition in plants (He and Ding, 2020; Shi et al., 2023). As a response to the low abscisic acid (ABA) levels in soybean seeds engineered to produce β-carotene (a precursor for ABA synthesis), changes in the expression of several genes encoding ABA-responsive transcription factors and FA desaturases resulted in an elevated OA/LA ratio while the levels of other FAs remained unchanged (Schmidt et al., 2015). External ABA application to Siberian apricot seeds resulted in an up-regulation of ABA-responsive genes and an increase in the OA/PA ratio (Huo et al., 2020). Increased levels of ABA and auxin in a protein kinase mutant resulted in increased OA levels, while the levels of PUFAs were decreased in *B. napus* seeds. The decreased sensitivity of this protein kinase mutant to ABA and glucose is suggestive of interactions between ABA and sugar signaling (Wang et al., 2022a). Auxin is also involved in modulating FA composition by regulating expression of FA desaturase genes in soybean as part of signaling and responses to heat and drought stress (He and Ding, 2020; Ozga et al., 2016; Ram Kumar et al., 2021). Foliar applications of auxin resulted in an increase in PA and OA levels in safflower plants under drought stress, a common response to lower membrane fluidity as in heat stress (Mousavi et al., 2022).

Here, we explored the genetic landscape of diverse soybean PIs for different FA compositions in the context of environmental stresses. Specifically, we conducted mGWAS to identify genomic regions linked to variations in FA compositions in 218 soybean PIs grown under field environmental conditions. Our focus was on the FA compositions and ratios relevant to both human and animal nutrition and compositional changes in membrane lipids and oil during abiotic and biotic stresses.

## 2 Materials and methods

### 2.1 Plant materials and environmental data

A total of 218 soybean PIs were selected from a diverse germplasm collection (Singer et al., 2022) and grown in Blacksburg and Warsaw in Virginia in 2020 and 2021, using a randomized complete block design. Each location had two similar plots, with each PI grown in both plots, allowing for two replicate measurements for each location and year. Collectively, eight seed samples were taken from each PI line. At each location, plants were grown in two 3-m, two-row plots with a 76-cm row spacing. Commercial cultivars Ellis and AG4403 were included at both locations as a reference for seed FA composition. Average daily temperature and precipitation data for the soybean growing seasons in Blacksburg and Warsaw in 2020 and 2021 were obtained from the National Oceanic and Atmospheric Administration’s (NOAA) National Centers for Environmental Information database (Menne et al., 2012). Least squares means were calculated, and Tukey’s HSD test was used to compare means at α = 0.05. Analysis of variance (ANOVA) was performed on monthly temperatures using a least squares model in JMP (JMP Pro, version 17.2.0, SAS Institute Inc.) with year, location, and year **·** location interaction as fixed effects.

### 2.2 FA and statistical analyses

A total of 1,744 seed samples were ground into a homogenous powder using a water-cooled 1095 KnifetechTM sample mill (Foss Analytical, MN, USA). Oil was extracted and FA levels were analyzed as described (Collakova et al., 2013). Briefly, 5 mg of dry powder for each seed sample was extracted using the Bligh and Dyer biphasic extraction method (Bligh and Dyer, 1959). Heptadecanoic acid was used as an internal standard to correct for % recovery for quantification purposes. FA methyl esters were prepared by acidic transesterification and analyzed on an Agilent 7890A gas chromatograph equipped with a flame ionization detector (GC-FID) as described (Collakova et al., 2013). Replicate seed samples from selected PIs and Ellis were also analyzed with GC coupled to an Agilent 5975C series single quadrupole mass spectrometer to confirm the identity of FAs using the NIST14 spectral library (National Institute of Standards and Technology, NIST).

Composition and ratios of different FAs were calculated using FA content data for each seed sample (n = 8 per PI line, n = 2 per PI line per each location and year combination). FA composition was expressed in two ways for each analyzed seed sample: (i) as a percentage of seed dry weight, reflecting seed FA composition with a sum of % of total FA as a proxy for the % seed oil and (ii) as a percentage of total FAs, approximating FA composition in oil. Then, Tukey’s HSD test was used to compare means of FA composition and FA ratios across all 218 PIs at α = 0.05. ANOVA and Principal Component Analysis (PCA) were performed in JMP (JMP Pro, version 17.2.0, SAS Institute Inc.) using FA composition in a least squares model with genotype, year, location, and year **·** location interaction as fixed effects and FA levels for each sample, respectively.

### 2.3 Genotypic data

Gene model and gene annotation information was obtained for the PIs used in this study from the SoyBase database (Brown et al., 2020). The genotype matrix was constructed using 42,291 SNPs from the SoySNP50K Illumina Infinium BeadChip (Song et al., 2013). Genotype data preprocessing was performed using base R (version 4.3.2) and RStudio (version 2023.9.1.494). SNP alleles were converted from alphabetic to numerical representations using a custom SNPtoNum() function, which encodes genotypes as {0, 1, 2} based on homozygous and heterozygous states. Monomorphic SNPs were identified using the sapply() function to check for columns with only a single unique value. Allele frequency (p) was calculated using colMeans() function, and minor allele frequency was determined using the pmin() function. After filtering out monomorphic SNPs and SNPs with a minor allele frequency of 0.05, 36,037 SNPs were retained for further analysis.

After filtering, the genomic relationship matrix was constructed from the resulting genotype matrix containing encoded SNP information. The genotype matrix (W) was centered using the scale() function from base R. The genomic relationship matrix (G) was derived following VanRaden’s method (VanRaden, 2008) after computing the cross-product of the centered genotype matrix (WW_t_) using the formula G = WW_t_/(2**·**sum(p(1-p))), where p is the frequency of the reference allele. Finally, the genomic relationship matrix was normalized to ensure proper scaling of the genetic variance component by dividing all elements by the mean of the diagonal elements. This procedure produces a genomic relationship matrix where the average diagonal element equals 1, scaling the genetic variance component for downstream analyses.

Best Linear Unbiased Estimates (BLUEs) for phenotypic traits were calculated using a weighted two-stage approach, which is common in multi-environment trials (Endelman, 2023; Verbyla, 2023; Möhring and Piepho, 2009). Briefly, trait values were modeled separately for each environment (year-location combination) using the model: trait ~ genotype + replicate. For each environment-specific model, reliability weights were calculated based on the residual variance and the number of replicates per genotype. BLUEs from individual environments were then combined using a weighted average approach, with weights proportional to their reliability. This approach accounts for potential heterogeneity in variance across environments while properly capturing genotype-by-environment interactions.

Narrow-sense heritability (*h* ^2^) was estimated by using a bootstrap approach with 1,000 resamples as described (Piepho and Mohring, 2007; Guidalevich et al., 2025). Mixed models were fitted with the genomic relationship matrix using restricted maximum likelihood (REML) within the mixed.solve() function from the GWAS package in R (RStudio, version 2023.9.1.494), calculating heritability as the ratio of additive genetic variance (V_A_) to total phenotypic variance (V_P_) where V_P_ = V_A_ + V_e_ and V_e_ represents the residual variance not attributable to additive genetic effects. The formula used was *h* ^2^=V_A_/(V_P_). Standard error (SE) was calculated as the standard deviation of bootstrap-generated *h* ^2^ values.

### 2.4 Metabolite genome-wide association study

mGWAS was performed using 44 different combinations of FA compositions and ratios. Compositions and ratios of different FAs were expressed as % of seed or % of oil using FA content data for each PI line. SNPs were then analyzed for the association with FA compositions or ratios using the GWAS() function from the rrBLUP package in R (RStudio, version 2023.9.1.494). Year, location, and block number were included as fixed effects to account for the variance attributed to environmental effects. A kinship matrix was included to account for relatedness between individuals and to reduce false positive associations due to population structure. Quantile-quantile (QQ) plots were generated for each trait by plotting the observed -log_10_(*p*) values against the expected -log_10_(*p*) values from a uniform distribution. The genomic inflation factor (λ) was calculated as the median of the χ^2^ distribution of observed *p*-values divided by the expected median χ^2^ value (Stich and Melchinger, 2009; Kaler et al., 2019).

To visualize the SNP *p*-values for each ratio, Manhattan plots were generated using the manhattan() function from the qqman() package (version 0.1.9) in R. The effective number of markers for each chromosome was calculated using the meff() function from the poolr package (version 1.1-1) (Li and Ji, 2005). Effective markers by chromosome were calculated to account for chromosome-specific linkage disequilibrium patterns between markers, then summed to obtain the total number of effective markers. This approach provides an accurate estimate of the number of independent tests, avoiding overly conservative multiple testing correction that would occur if treating all markers as independent. The sum of effective markers was used to determine a significance threshold for selecting significant SNPs with 95% confidence as described (Singer et al., 2022). Briefly, the significance threshold was determined using the formula: threshold = 1 - (1 - α)^(1/M_eff_), where M_eff_ represents the total number of effective markers (5,067) and α is the desired significance level (0.05 in this case).The resulting significance threshold was found to be approximately 1.01 **·** 10^-5^. A suggestive threshold was also set using 75% confidence (α = 0.25) and was determined to be approximately 5.68 **·** 10^-5^.

SNPs with *p*-values above the suggestive threshold were further analyzed to extract effect sizes (β) and SEs. For each SNP, a mixed linear model that mirrored the model set by the GWAS() function was fitted. β coefficients were estimated using mixed model solving using the mixed.solve() function, with the coefficient representing the change in the trait value associated with one copy of the alternative allele. SEs were calculated to assess the precision of these effect estimates. To reduce potential redundancy and identify independent significant loci, a post-GWAS clumping procedure was performed using a custom R function. SNPs were clustered within a 250-kb window and grouped based on linkage disequilibrium (r^2^ threshold of 0.5) (Misra et al., 2017). For each chromosome, lead SNPs and their correlated neighboring SNPs were identified, keeping the most significant SNP as the representative for each linkage group. This approach helps to minimize the reporting of highly correlated SNPs and provides a more concise representation of the genetic associations with FA compositions. Finally, candidate genes were identified from the gene models for Williams 82 (Glyma.Wm82.a2.v1 genome) available in the SoyBase database (Brown et al., 2020) within 10-kb flanking regions of the significant or suggestive SNPs. 10-kb regions are commonly used for identifying genes in soybean GWAS (Ayalew et al., 2022; Ravelombola et al., 2021; Steketee et al., 2020).

## 3 Results

### 3.1 Environmental differences between locations and years

**Figure 1:**
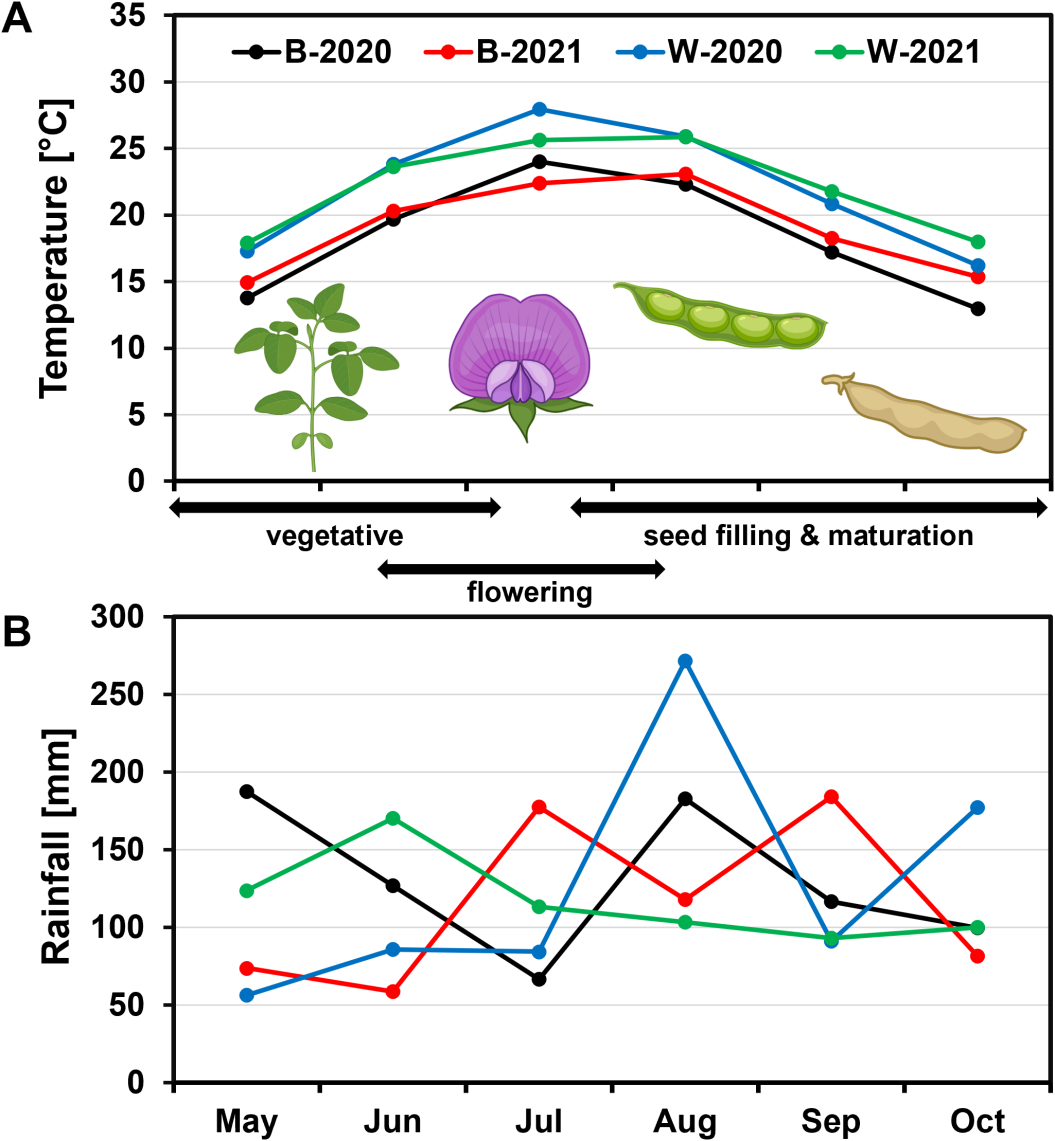
Environmental data for the soybean growing seasons in Virginia. **(A)** Average monthly temperatures and **(B)** cumulative rainfall per month are shown in the context of key stages in soybean growth and development. In both 2020 and 2021, soybean seeds were planted in Blacksburg (B) and Warsaw (W) in May and mature pods harvested in October. Approximate time ranges for different soybean developmental stages are indicated by double arrows. Environmental data for this period was collected from NOAA’s National Center for Environmental Information (Menne et al., 2012) for both locations and years. Images of different parts of a soybean plant were licensed from BioRender.

Heat and drought are known to affect FA composition in oilseeds (Bellaloui et al., 2015; Hernández et al., 2019; Narayanan et al., 2020; Sehgal et al., 2018). To evaluate potential differences in environmental conditions between the two locations and years that could affect seed FA composition, average temperatures and rainfall from the NOAA National Centers for Environmental Information database (Menne et al., 2012) were compared for the two locations and years during the growing season. Average season-long temperatures followed similar trends in both locations. However, Warsaw is a warmer location than Blacksburg, and the average monthly temperatures were higher there than in Blacksburg regardless of the year (**Figure 1A**). These temperature differences were statistically significant (Tukey’s HSD test, α < 0.05) for most months (**Supplementary Table S1A**). Average daily temperatures were the highest in Warsaw in July 2020 when soybeans flower and initiate seed development (**Supplementary Table S1B**). Year 2020 was the rainiest for both locations (**Supplementary Table S1A**). As expected, rainfall did not follow any specific trend during both growing seasons (**Figure 1B**), and there were variations in cumulative precipitation by month across years and locations with the highest cumulative monthly precipitation seen in Warsaw in August 2020 (**Supplementary Table S1A**) due to several rainy days (**Supplementary Table S1B**). There was a drought period in Warsaw from May to July in 2020 and in Blacksburg from May to June in 2021 (**Supplementary Table S1B**). Overall, the Warsaw 2020 growing season was the most different compared to other seasons during the seed development period. Collectively, these environmental variations happening during the reproductive stages of soybean seed development created environments that could impact FA composition in soybean seeds.

### 3.2 Effects of genotype, location and year on variance in soybean FA composition

**Figure 2:**
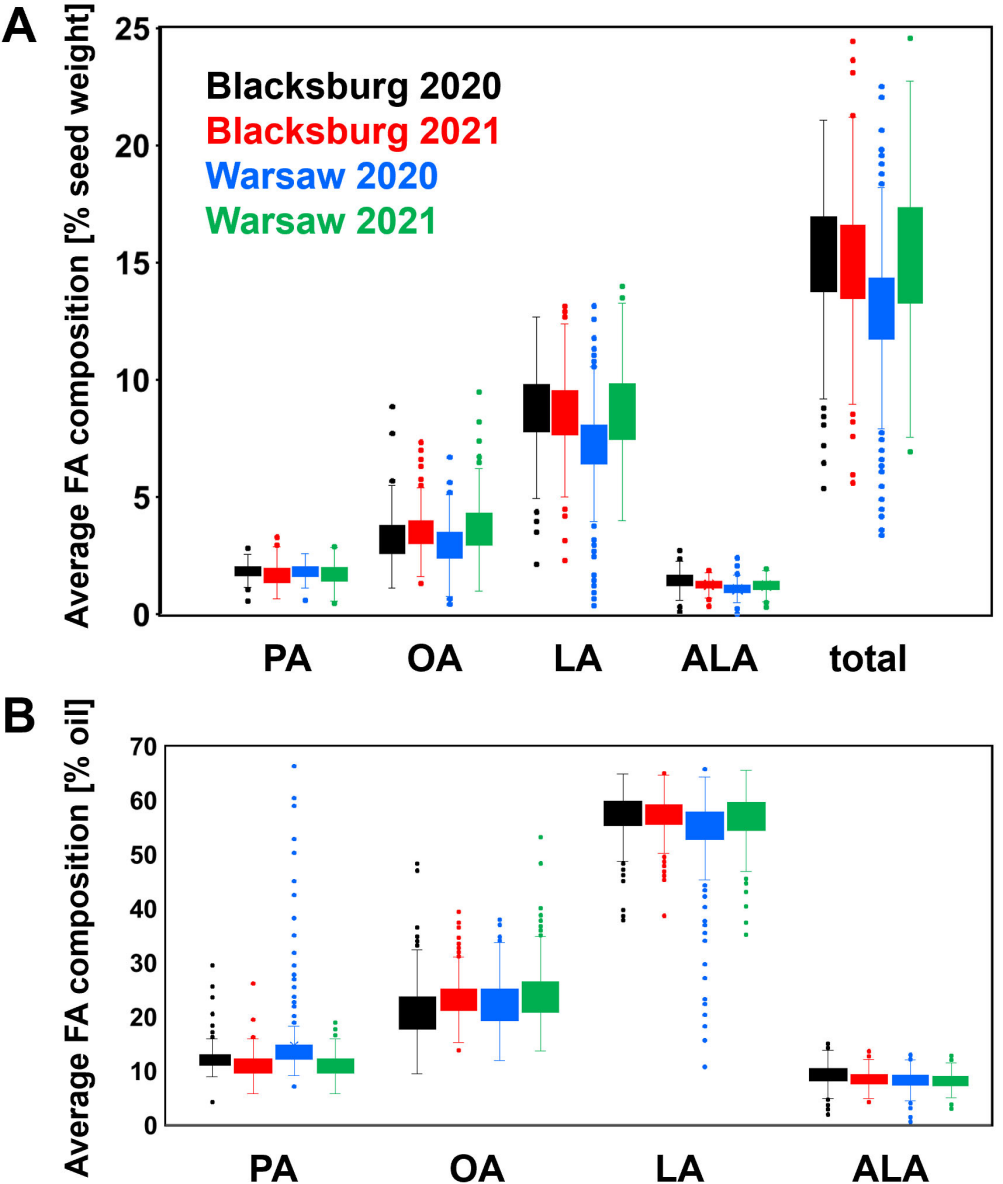
Average FA compositions in 218 soybean PIs grown in two locations and two years in Virginia. Soybean PIs were grown in a randomized block design in Blacksburg and Warsaw in 2020 and 2021 and FA analysis was performed as described in Materials and methods. FA composition was expressed as FA % of seed weight and FA % in oil of individual PIs and shown as an average ± SD of all tested PIs grown at each location and year with two replications. **(A)** Average seed FA composition and **(B)** Average oil FA composition. Based on ANOVA, no statistically significant differences (*p* < 0.05) were identified among the specific FAs in the PIs from both locations and years.

To obtain statistically significant results, GWAS requires sufficient differences in phenotypic data for correlating SNPs with specific phenotypes, which makes evaluating the variance in FA composition data essential. Considering the low genetic diversity in soybeans, we first investigated if different environmental conditions affected FA composition in diverse genetic backgrounds of soybean PIs in a global way. Averaging composition of the individual FAs from 218 PIs revealed that seed FA composition and ratios were similar regardless of the location, year, and the method used for expressing FA composition, though there was a high degree of variance (**Figure 2**; **Supplementary Table S2**). Tukey’s HSD test was also conducted to determine if there were statistically significant differences (α = 0.05) in the means of seed FA compositions (**Table 1**) and ratios (**Supplementary Table S3**) for different locations and years. Overall, seeds of soybean PIs grown in Warsaw in 2020 showed the most statistically significant differences for most FA compositions and ratios, particularly favoring the high PA/LA ratio, relative to the other location and year combinations regardless of how FA composition was expressed. As for the individual FAs, OA composition for both OA as % seed weight and % oil varied the most and was significantly different for all four combinations of locations and years (**Table 1**). There was an expected degree of variance for this type of data when levels or % of specific metabolites are averaged in genetically diverse lines without considering potential differences due to genotypes.

**Table 1:** Statistical analysis of means of FA composition in soybean PIs grown in two different locations and years in Virginia.

FA	% of seed weight				% of oil			
	Blacksburg		Warsaw		Blacksburg		Warsaw	
	2020	2021	2020	2021	2020	2021	2020	2021
PA	1.85 A	1.67 B	1.81 A	1.70 B	12.3 B	11.1 C	14.7 A	11.0 C
OA	3.23 C	3.52 B	2.98 D	3.69 A	21.1 D	23.3 B	22.8 C	24.0 A
LA	8.74 A	8.61 A	7.17 B	8.69 A	57.2 A	57.1 A	54.4 B	56.9 A
ALA	1.43 A	1.28 B	1.07 C	1.23 B	9.38 A	8.53 B	8.13 C	8.14 C
Total	15.3 A	15.1 A	13.0 B	15.3 A	N/A	N/A	N/A	N/A

Soybean PIs were grown in Blacksburg and Warsaw, VA in 2020 and 2021 and their dry seeds analyzed for FA levels as described in Materials and Methods. Least squares means were calculated for both types of FA composition (% seed weight and % oil) and used for the Tukey’s HSD test. N/A, not applicable.

To include genotypes and potential location and year interactions, we also explored the fixed effects of these different factors on FA composition in soybean seeds by using both types of FA composition data. Specifically, we used ANOVA to investigate if genotype, location, year, and year **·** location interaction had significant effects on FA composition in the seeds of soybean PIs by comparing least squares means. ANOVA revealed significant effects for all fixed factors for most FA compositions and ratios (**Supplementary Table S4**). Only some factors had no statistically significant effects on specific FA traits and included the effects of: (i) location on PA and OA composition (% seed weight) and on the OA/PA ratio, (ii) year on ALA (% seed weight) and the OA/ALA and (OA+ALA)/LA ratios, and (iii) the interaction of year **·** location on the OA/(PA+ALA) and (OA+ALA)/(LA+PA) ratios.

**Figure 3:**
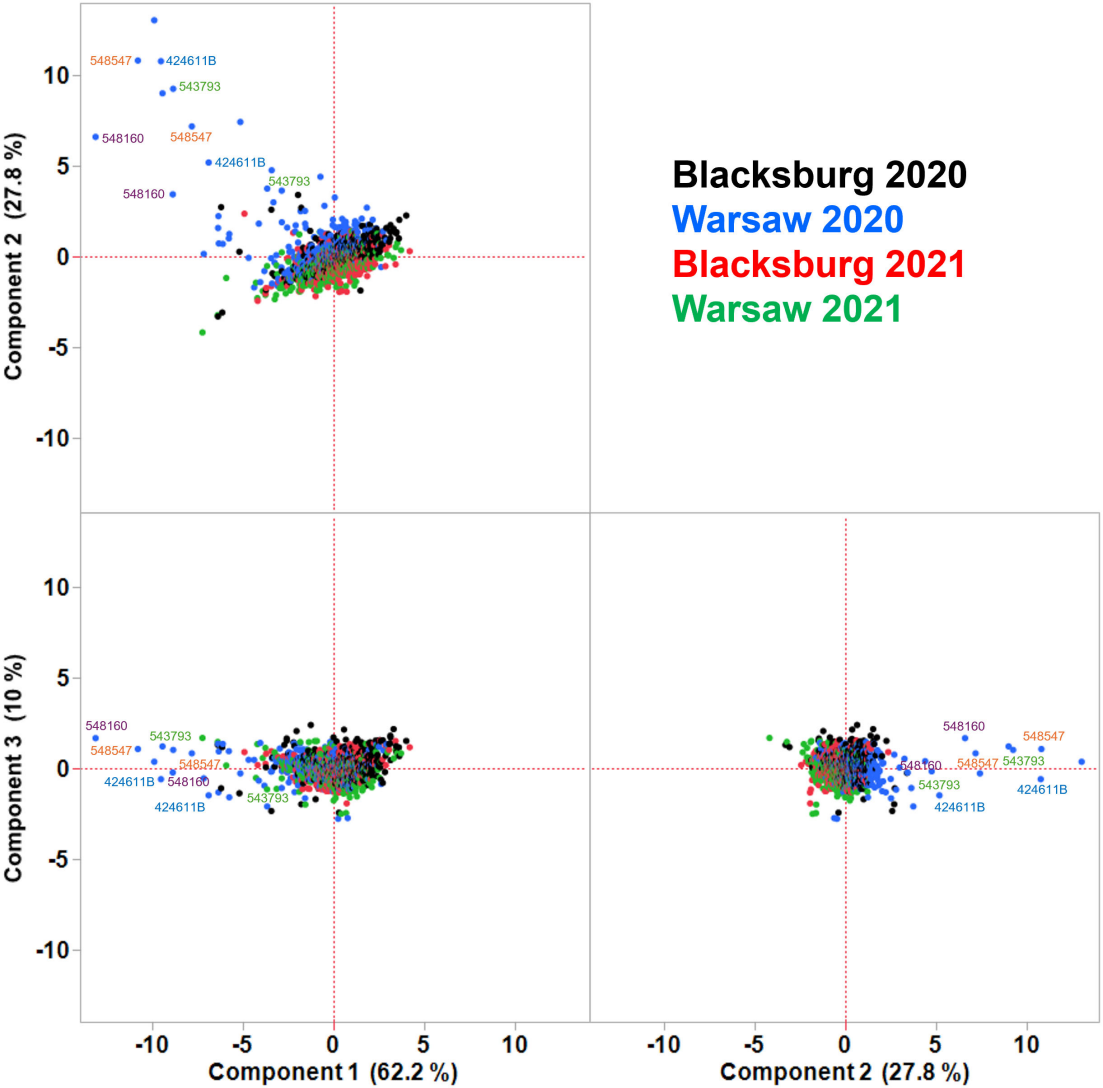
PCA on correlations of oil FA composition of PIs from two different locations in Virginia and years. Soybean PIs were grown, and their seed FA content analyzed as for **Figure 1**. Most PIs cluster together regardless of location or year, suggesting their oil FA compositions are similar. PC 1 and 2 account for nearly 90% of variance and explain the differences in oil FA composition in some PIs, particularly those grown in Warsaw in 2020. Although most PIs grown in Warsaw in 2020 cluster with the rest of PIs, many are outliers at the borderline of being separated from the main cluster. The four PIs with both replicates as outliers are shown in different colors.

To further evaluate the variance in these datasets (**Supplementary Table S2**) in the context of specific genotypes and to identify the PIs contributing the most to this variance, PCA was performed on both types of FA composition datasets. This multivariate statistical method is used to evaluate if samples are similar or different from each other based on specific traits. Here, we used PCA to assess whether there is sufficient variance among the PIs based on FA composition due to different environmental conditions for mGWAS. PCA confirmed the lack of major differences in FA composition regardless of location or year in many PIs (**Figure 3**; **Supplementary Figure S1**). Based on the PCA results, many PIs from all four locations and year combinations clustered together, suggesting that these PIs were not different from each other in terms of FA composition. However, some distinction between two years can be observed based on the clustering of PIs from different locations and the same year. This is evident from the overlapping blue and black dots representing some PIs from Blacksburg and Warsaw in 2020 that are separated from overlapping green and red dots representing some PIs from the two locations in 2021 (**Figure 3**; **Supplementary Figure S1**). In addition, many outliers, particularly for the seed-based FA composition, were identified (**Supplementary Figure S1**). PCA on the oil-based FA composition showed only several extreme outliers and specifically for PIs grown in Warsaw in 2020, with only four PIs with both replicate measurements as outliers. This was evident from the separation of PI424611B, PI543793, PI548160, and PI548547 from the rest of the PIs based on PCs 1 and 2, but not PC3 and several other outlier PIs with another replicate clustering with the rest of the PIs (**Figure 3**). It appears that these PIs responded consistently to different environmental conditions specifically in Warsaw in 2020 compared to the remaining plants. Overall, there may be enough PIs with environmentally induced differences in FA seed composition for mGWAS.

### 3.3 Metabolite genome-wide association study

Our goal was to identify SNPs associated with genes encoding proteins likely regulating FA composition in soybean seeds through stress-related signaling pathways. We expected regulatory proteins to be responsive to the environment and have weaker effects on FA composition in soybean seeds than metabolic enzymes (FA elongases and desaturases), which would be reflected by weaker associations of SNPs with the corresponding genes. Therefore, to increase the chances of identifying SNPs, suggestive in addition to statistically significant *p*-value cutoffs were used. All combinations of complex FA ratios regardless of any relevance to biological processes and/or practical applications were also included to help with SNP identification. Because of the rather large environmental influence on these FA phenotypes, we also estimated the relative contribution of genetic and environmental variance to the observed FA phenotypes by determining the narrow-sense heritability for each FA composition and ratio. The heritability ranged from 0.015 to 0.7 for FA ratios; low heritability was observed for several FA ratios involving PA and high heritability for ALA-related FA ratios (**Supplementary Table S5**). FA ratios relevant to abiotic and biotic stresses such as PA/(OA + LA + ALA) and (PA + OA)/(LA + ALA) had low heritability.

mGWAS was performed using 44 different combinations of FA compositions and ratios at the significant (*p* < 10^-5^ at 95% confidence) and suggestive (*p* < 5.68 **·** 10^-5^ at 75% confidence) *p*-value cutoffs to identify 20 and 90 SNPs for 21 and 36 FA composition and ratio phenotypes, respectively (**Supplementary Table S6**). Many of the identified SNPs were associated with more than one FA composition and/or ratio that included these FAs in a way that a particular FA was present in every related FA ratio within that group. This FA was viewed as specific to the corresponding SNP and FA ratio group. In addition, many of the complex FA ratios represent similar ratios and can be considered redundant. Collectively, these 93 SNPs were located within 10-kb regions containing 124 unique genes and some of them were linked (17 different linkage groups of at least two SNPs), while 34 SNPs were independent (**Supplementary Table S7**). Because SNPs adjacent to the SNP associated with the specific phenotype are inherited together and form a linkage group, focusing on linked SNPs is expected to increase chances of finding the genes responsible for the observed metabolic phenotypes. Therefore, our focus was mostly on significant and suggestive linked SNPs for composition of individual FAs. As expected, some SNPs were associated with an increase, while the others with a decrease in the corresponding FA traits, as evident from their respective β coefficients (**Supplementary Table S8**).

**Figure 4:**
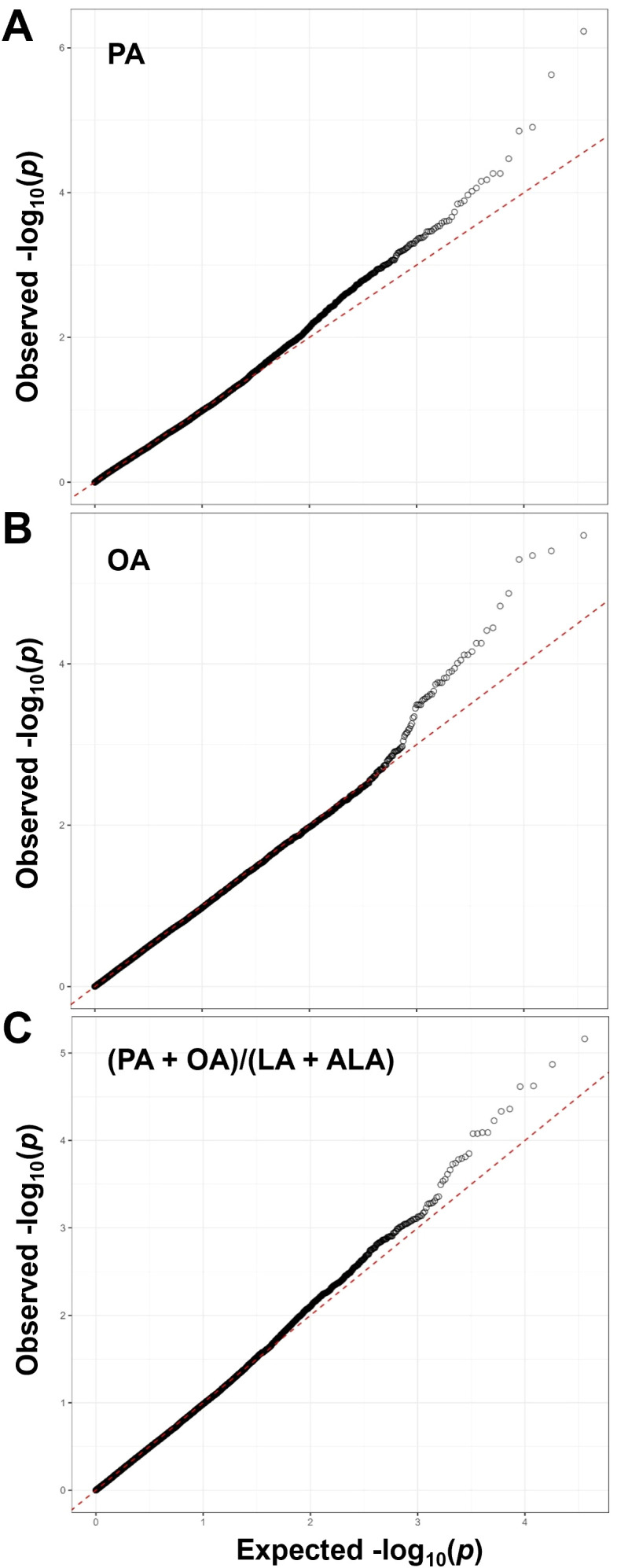
QQ plots for the selected FA composition and ratio traits. **(A)** Oil-based PA composition, **(B)** Oil-based OA composition, and **(C)** The (PA + OA)/(LA + ALA) ratio. The QQ plots for these selected FA traits were generated by plotting the observed and expected -log_10_(*p*) values from a uniform distribution and the corresponding λ factors were calculated as described in Materials and Methods. False positives and negatives were properly controlled as evident from the QQ curve shapes and λ factors (0.989, 0.985, and 0.969 for the PA, OA, and (PA + OA)/(LA + ALA) ratio, respectively).

**Figure 5:**
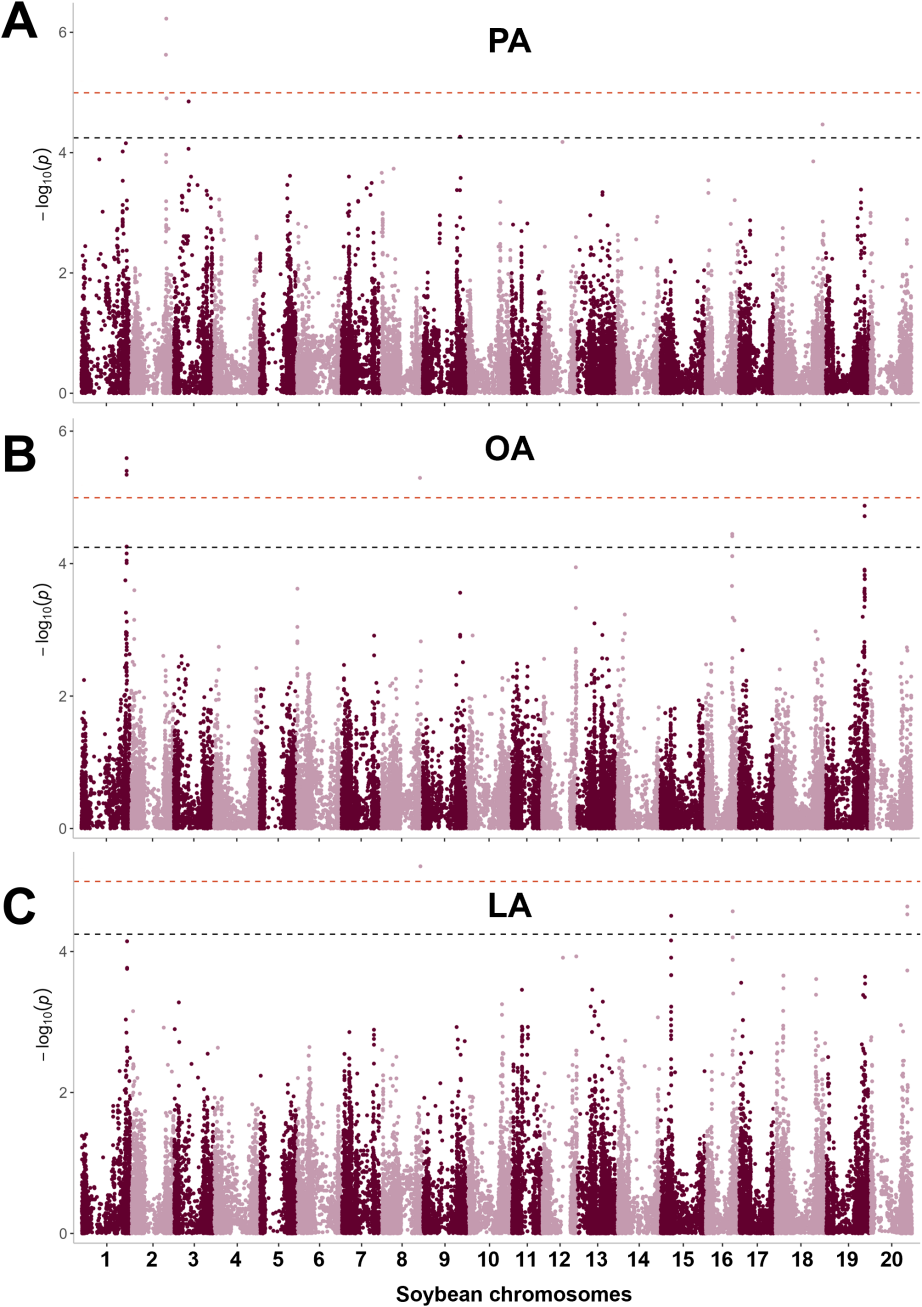
Manhattan plots showing significant and suggestive SNPs for FA composition as a percentage of oil. SNP associations are shown as the negative logarithm of the *p*-values obtained from the GWAS model. Alternating dark red and light red points show the localization of SNPs on different chromosomes. Orange and black lines show the significance thresholds for significant (*p*-value < 10^-5^) and suggestive (*p*-value < 5.68 **·** 10^-5^) SNPs, respectively. For PA **(A)**, significant and suggestive SNPs were identified on chromosomes 2, 3, 9, and 18, while for OA **(B)**, they were located on chromosomes 1, 8, 16, and 19. **(C)** LA-associated SNPs were found on chromosomes 8, 15, 16, and 20. SNPs ss715602319 and ss715624375 on chromosomes 8 and 16 were the same as for OA.

**Figure 6:**
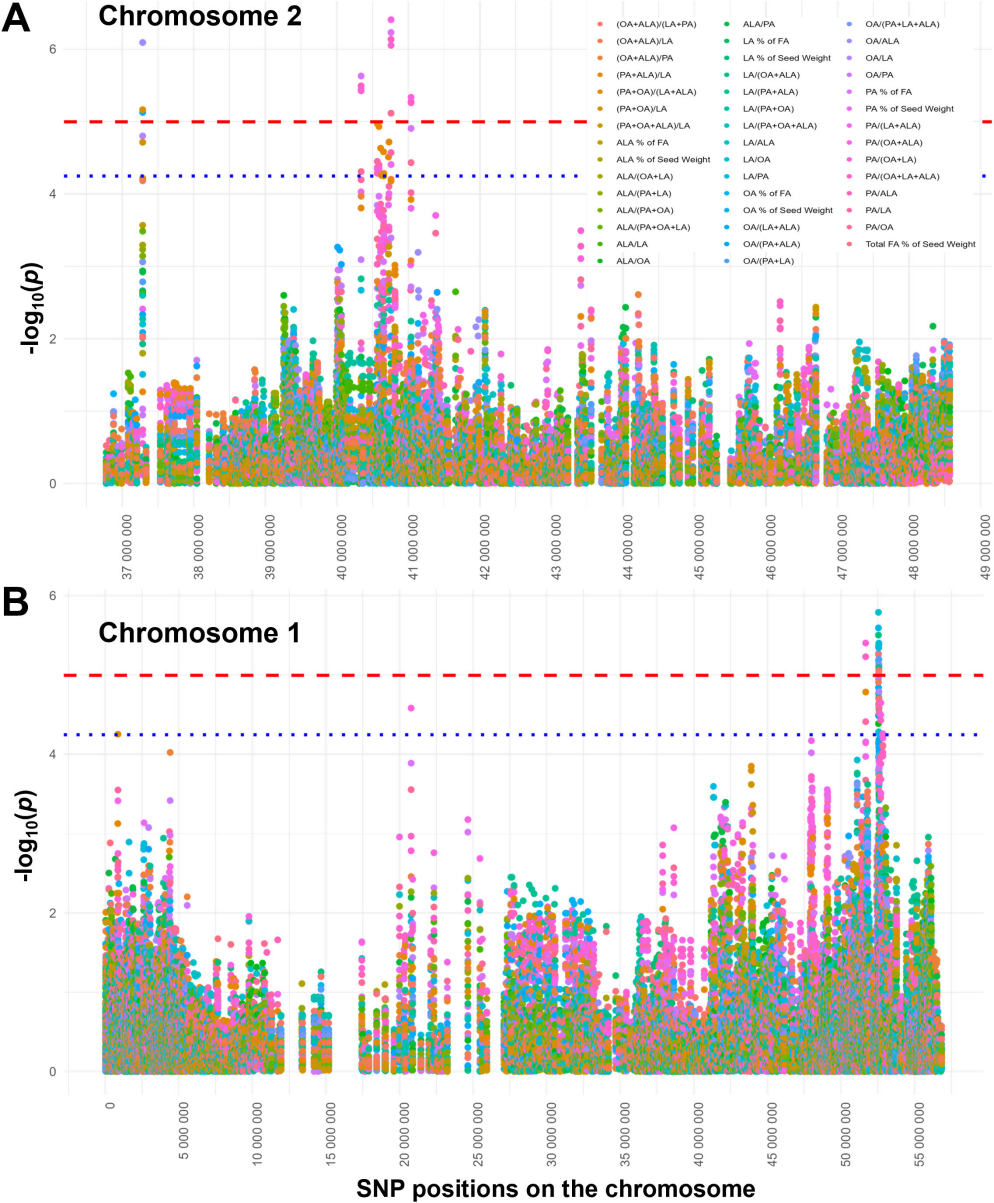
Manhattan plots showing significant and suggestive SNPs for selected chromosomes and multiple simple and complex FA ratios. SNP associations and significance levels (also represented by the orange and blue lines for significant and suggestive SNPs, respectively) are the same as in **Figure 4**. SNPs associated with individual FA ratios are color coded. Only the last third of chromosome 2 **(A)** is shown as no significant or suggestive SNPs were found on this chromosome before 35 Mbs. Pink, purple, and mustard colors for the clusters of significant and suggestive SNPs between 40 and 41 Mbs correspond to the SNPs for several PA-related FA ratios, including (PA + OA)/(LA + ALA). **(B)** For chromosome 1, pink and blue color for the significant and suggestive linked SNPs between 50 and 55 Mbs correspond to PA- and OA-related FA ratios.

To ensure that our models appropriately control false positives and negatives, QQ plots were generated and λ calculated for each FA trait. The resulting QQ plots for oil-based PA and OA composition, and for the (PA + OA)/(LA + ALA) ratio suggest the existence of significant SNPs associated with these FA traits (**Figure 4**). In all cases, λ was close to 1 and most QQ plots for the remaining FA compositions and ratios had the expected straight line until reaching the suggestive and significant *p*-value thresholds where the observed probability was higher than the expected one (**Supplementary Figure S2**). QQ plots for seed-based FA composition and some FA ratios, particularly those containing ALA in the nominator were indicative of the lack of significant or suggestive SNPs. Consistent with the QQ plots, no significant SNPs were identified for any FAs when FA composition was expressed as a percentage of FA content per seed weight and for ALA when ALA composition was expressed as a percentage of ALA in oil. However, several significant, mostly unlinked SNPs were identified for PA, OA, and LA with oil-based FA composition and specific FA ratios, and the largest linkage groups were found on chromosomes 1, 2, and 19 (**Figures 5**, **6**, **Supplementary Figures S3**, **S4**, **Tables S6**, **S7**).

Two major linkage groups were identified for PA (2.3 and 3.4, where the first number represents the chromosome and the second one the linkage group; see **Supplementary Table S7**). In linkage group 2.3, nine linked SNPs (ss715582608 - 44) were associated with eight genes encoding regulatory and metabolic proteins and were suggestive for the elevated PA/LA and (PA + ALA)/LA ratios with PA as the FA driving these genetic associations. One of the metabolism-related genes (*Glyma.02g218700*) near SNP ss715582631 encodes a glycerol-3-phosphate dehydrogenase known to influence FA composition towards FA unsaturation in soybean seeds (Zhao et al., 2021). Near this group of linked SNPs, two individual SNPs (ss715582649 and ss715582700) were also significant for high PA as a % of oil and many other FA ratios involving PA and OA and associated with a methyl transferase or a transporter gene and two uncharacterized genes. Five metabolic and regulatory genes (*Glyma.03g229300* - *Glyma.03g229600*) were associated with a group of SNPs (ss715586385 – 9) that were related to elevated PA/OA ratios and were part of linkage group 3.4 (**Supplementary Table S7B**).

For OA as a % of oil, four major linkage groups (1.4, 1.5, 16.1, and 19.2) were identified (**Supplementary Table S7**). In the adjacent linkage groups 1.4 and 1.5, three significant and seven suggestive SNPs (ss715580258 – 60 and ss715580261 - 315), respectively, were also associated with several complex FA ratios like the OA/(LA + ALA) ratio in group 1.4. The high OA/(LA + ALA) ratio is of interest as it reflects the MUFA/PUFA ratio relevant to human and animal nutrition. The β coefficients were always positive for the OA/LA-related ratios and always negative for LA/OA-ratios (**Supplementary Table S8**), conferring to the increased simple and complex MUFA/PUFA ratios. These OA-associated SNPs were near or within 15 adjacent metabolic and regulatory genes (*Glyma.01g190700* - *Glyma.01g194200*). However, information on specific biological processes these genes are involved in is not available, except for the strigolactone biosynthetic gene *Glyma.01g191200* (Liu et al., 2020). Similarly, linkage group 16.1 is also associated with various OA/LA-related ratios, including OA/(LA + ALA). In this case, the OA/LA-related ratios are low, as indicated by the negative β coefficients for these ratios (**Supplementary Table S8**). Group 16.1 contains four linked SNPs (ss715624374 - 8) associated with four adjacent genes (*Glyma.16g151000* - *Glyma.16g151300*). *Glyma.16g151000* encodes a protein that binds phospholipids, while *Glyma.16g151200* syntaxin involved in vesicle trafficking. TatD-related DNAse and aquaporin AQP67 are encoded by *Glyma.16g151100* and *Glyma.16g151300* and these types of proteins are known to be involved in stress responses (Dasmandal et al., 2020; Feng et al., 2019). However, AQP67 (designated as the stress-induced protein GmSIP1;2) was shown as unresponsive to drought stress (Feng et al., 2019).

The OA- and LA/OA-driven linkage group 19.2 contains eight SNPs (ss715635406 - 22). Group 19.2 is also associated with several low OA/LA-related ratios, including OA/(LA + ALA). There are nine consecutive genes (*Glyma.19g192600* - *Glyma.19g194300*) associated with the SNPs within this linkage group. This group of genes was also identified by GWAS to be involved in phosphorus efficiency in maturing soybean plants (Wang et al., 2022b). In Arabidopsis, homologs of these genes are either developmental or related to phosphate metabolism (Cerise et al., 2023; Jung et al., 2009; Muller et al., 2007; Skinner and Gasser, 2009), though *Glyma.19g193800* encodes an AN1-type zinc finger protein that could be drought stress-related (Ding et al., 2013). For low LA as a % of oil, only a small linkage group (20.1) of two suggestive SNPs (ss715638312 and ss715638314) associated with four genes of unknown or unrelated biological functions was identified. *Glyma.20g187000* is a developmental gene involved in plastidic RNA editing and chloroplast function (Zhu et al., 2020).

The (PA + OA)/(LA + ALA) ratio is related to compositional changes in FAs when the levels of PA and OA increase at the expense of ALA and LA to decrease membrane fluidity as part of adaptation to drought and heat stress (Henschel et al., 2024; Higashi and Saito, 2019; Sharma et al., 2023). This complex FA ratio was not well represented in our data, but it was increased for all relevant SNPs (**Supplementary Table S8**). This FA ratio is associated with only two linkage groups (15.1 and 19.1) and two individual SNPs (ss715582171 and ss715627896). The first SNP is not near any gene, while the second one is in vicinity of three regulatory genes, one of which (*Glyma.17g053700*) encodes a heat-shock transcription factor. For 15.1, three suggestive SNPs (ss715620394, ss715620407, and ss715620417), are related to the (PA + OA)/(LA + ALA) ratio. There was only one such SNP (ss715635104) linked to two uncharacterized genes in group 19.1. SNP ss715620394 is associated with DNA-J domain chaperone protein encoded by *Glyma.15G146900* and ss715635104 with two uncharacterized proteins.

## 4 Discussion

### 4.1 Environmental influence on FA composition in soybean seeds

Soybean domestication has led to a significant loss of genetic diversity during the past 9,000 years, leading to relatively stable FA composition in the seeds of different soybean cultivars and breeding lines (Derbyshire et al., 2023; Sedivy et al., 2017; Song et al., 2023). As expected, seed FA composition was consistent across most tested soybean PIs regardless of the location and year, with LA predominating and followed by OA, PA, and ALA, which is consistent with previously reported FA composition values (Abdelghany et al., 2020; Di et al., 2022; Messina, 2016; Song et al., 2023). Although seed FA composition was stable in the majority of soybean PIs, environmental conditions were sufficiently different, especially in Warsaw in 2020 and between the two years, and coincided with soybean reproductive stages to induce changes in FA composition in many PIs. The observed higher PA and lower PUFA levels could be related to higher temperatures and lower rainfall during key stages of soybean seed development in Warsaw 2020. However, the two locations and years likely vary in other environmental aspects (occurrence and interactions of other abiotic and biotic stressors) that were not accounted for in our study and could have had an impact on FA levels and composition. The important point is that the environmental conditions varied enough during key stages of soybean seed development to induce expected changes in seed FA composition so that statistically significant genotype-to-phenotype relationships could be identified by mGWAS.

The unsaturation level of FAs can be influenced by various environmental stresses, including drought and heat. Double bonds in *cis* configuration create kinks in the FA chains disrupting hydrophobic interactions between FAs within biological membranes, thus increasing membrane fluidity, which is not desirable at high temperatures (Rustgi et al., 2021). A common stress response during drought and heat stress includes lowering the unsaturation level by increasing the proportion of SFAs and sometimes also monounsaturated FAs such as OA at the expense of PUFAs such as ALA and LA (Schmidt et al., 2015; Mousavi et al., 2022; Wang et al., 2022a). Seeds from PIs grown in Warsaw 2020 showed the lowest levels of PUFAs and the highest levels of PA. Similarly, higher levels of PA and lower levels of OA were detected in the seeds of PIs from Blacksburg in 2020 than seeds from Blacksburg 2021, which could be attributed to the higher temperatures and lower rainfall in Blacksburg in July 2020. However, soybean PIs grown in Blacksburg in 2020 had the highest seed ALA levels, suggesting that other environmental factors that were not accounted for may have been interacting and affecting FA composition in an unpredictable way.

The molecular mechanisms driving membrane remodeling in terms of FA composition are complex and involve various metabolic and signaling pathways, including, but not limited to reduced expression of genes encoding FA elongases and desaturases for *de novo* FA synthesis (Hernández et al., 2019; Nahar et al., 2016; Narayanan et al., 2020) and lipid and FA exchange between membrane lipids and oil (Kanai et al., 2019; Bates, 2016; Bhandari and Bates, 2021). During seed filling, these processes occur naturally in soybean regardless of stress that a plant might be experiencing as diacylglycerol and FAs from membrane lipids are used for oil assembly to keep up with high demands of carbon for oil synthesis (Kanai et al., 2019; Bates, 2016; Bhandari and Bates, 2021). During this time, FA composition in oil will depend on the type of stress and the required response to overcome it. Other protective mechanisms, including up-regulation of stress-related genes and production of protective metabolites involving different types of regulatory mechanisms, are also in place (Zhang et al., 2015; Gelaw and Sanan-Mishra, 2021; Haak et al., 2017; Punzo et al., 2020; Razi and Muneer, 2021; Zandalinas and Mittler, 2022).

We expected the genotype-phenotype associations for regulatory and signaling genes involved in modulating FA composition to be weaker than for metabolic genes directly involved in FA elongation and desaturation. In addition, domesticated soybeans have relatively low genetic diversity, and many traits can be highly influenced by the environment. As expected, the heritability of FA ratios relevant to heat and drought stress such as PA/(OA + LA + ALA) and (PA + OA)/(LA + ALA) was low, suggesting that these traits are influenced more by environment than genetics. Using all possible FA ratios proved useful, as we were able to identify several significant and suggestive SNPs for different FA compositions and simple and complex FA ratios. In many cases, a SNP was associated with more than one FA ratio, suggesting that these ratios were at least partially redundant. In the case of PA, OA, and to some extent LA, a clear prevalence of these FAs was observed in groups of these partially redundant FA ratios associated with specific linked SNPs, suggesting that these individual FAs were driving the statistical differences in these ratios for mGWAS.

### 4.2 Metabolic genes influencing FA composition

Identification of metabolic genes that are known or implicated to affect soybean seed FA composition directly or indirectly can serve as a type of validation for our approach. Since we identified such genes, our approach is suitable for the identification of other genes potentially involved in modulating FA composition in a response to different environmental conditions. As part of the central carbon metabolism, plastidic glycerol-3-phosphate dehydrogenase (GmGPDHp1) and glucose-6-phosphate 1-dehydrogenase 1 (GmG6PDH1) produce carbon substrates and reductant (NADPH) used for FA synthesis and desaturation during seed development when light may be limiting (Allen et al., 2009; Hu et al., 2023; Zhao et al., 2021). These genes were identified as candidate genes based on several SNPs that were strongly associated with PA, LA or OA and the related FA ratios that would lower FA desaturation consistent with drought and heat stress. Overexpression of the GmGPDHp1 gene resulted in an overall increase in the levels of unsaturated FAs, particularly OA, thus altering an overall FA composition towards unsaturation in soybean seeds (Zhao et al., 2021). NAD kinases could also be part of this mechanism, as they control the NAD(H) to NADP(H) ratio, therefore contributing to the maintenance of redox homeostasis in plants under various abiotic and biotic stresses (Li et al., 2018). Activities of the plastidic dehydrogenases could rely on the availability of NADP produced from NAD+ by these NAD kinases. For the LA-driven FA ratios, the SNP ss715612955 is associated with *Glyma.12g234000* encoding a putative NAD kinase potentially involved in modulating FA composition through this mechanism.

Interestingly, the *GmG6PDH1* gene was associated with the same groups of linked SNPs as *Glyma.03g229600* encoding an uncharacterized protein homologous to the Arabidopsis *At1g09390* gene encoding a zinc finger FYVE domain-containing GDSL lipase/acylhydrolase (Lai et al., 2017). These enzymes either hydrolyze lipids to diacylglycerol and free FAs or attach FAs to lipids and have been associated with high-oil phenotypes in soybean seeds and maize kernels (Lai et al., 2017; Akoh et al., 2004; Liao et al., 2024; Luo et al., 2023). GDSL lipases/acylhydolases have potential to modulate the desaturation level in oil depending on the desaturation degree of membrane lipid-derived diacylglycerols and which FAs are available for oil synthesis. However, they can also have non-lipid substrates and specific metabolic functions of *At1g09390* and *Glyma.03g22960*0 gene products remain to be determined. The *Glyma.16g151000* gene encoding a phosphatidylethanolamine-binding protein is associated with two SNPs predicted to lower OA composition and OA-related FA ratios. Different alleles of this gene were shown to be associated with regulating flowering time (Chen et al., 2020; Liu et al., 2022; Ogiso-Tanaka et al., 2019). Differences in flowering time could shift the timing when plants are exposed to different stresses during membrane remodeling, FA synthesis, and oil assembly as part of seed filling, which could lead to differences in FA composition. Dehydrogenases and membrane-modifying enzymes represent molecular components of two different mechanisms contributing to modulating FA composition.

### 4.3 Effects of abiotic and biotic stress signaling on FA composition

In addition to regulating many diverse aspects of plant growth, development, and abiotic and/or biotic stress responses (Duhan and Pasrija, 2025; Gupta et al., 2024), the phytohormones ABA, auxin, ethylene, JA, and SA are involved in regulating FA and lipid metabolism (Bajguz and Piotrowska-Niczyporuk, 2023; Waadt et al., 2022; Yi et al., 2024). Interactions of the corresponding signaling pathways in plants under combinations of abiotic and biotic stresses are complex and can be synergistic or contradictory (Gupta et al., 2024; Singh and Roychoudhury, 2023; Waadt et al., 2022). Except for ethylene, which has opposite effects on FA composition (Li et al., 2023), these phytohormones were shown to alter PA, OA, and/or LA composition towards elevated PA and OA levels in leaves during drought and heat stress or in developing seeds of various species (Ghassemi-Golezani and Farhangi-Abriz, 2018; Huo et al., 2020; Wang et al., 2022a), including soybean (Ram Kumar et al., 2021; Schmidt et al., 2015). These FA composition changes are associated with changes in expression of genes encoding ABA-responsive genes and FA desaturases (Ghassemi-Golezani and Farhangi-Abriz, 2018; Ram Kumar et al., 2021; Schmidt et al., 2015; He and Ding, 2020). Similarly, aphid feeding is also accompanied by significant increases in the ratios of PA and OA to LA and ALA ratio, lowering the levels of ALA that can be used for JA synthesis as a mechanism to block JA signaling in soybean leaves (Kanobe et al., 2015).

Therefore, there appears to be a common trend of increasing the levels of FAs that have no and/or low degree of unsaturation such as PA and OA at the expense of the PUFAs LA and ALA during abiotic and biotic stresses, such as drought, heat, and herbivory. We were able to identify several SNPs associated with the increased (PA + OA)/(LA + ALA), and the related PA/(LA + ALA) and/or OA/(LA + ALA) ratios that represent these types of changes in FA composition, with primarily PA and/or OA as the driving factors for significance of these FA ratios. These SNPs were within or near the genes encoding proteins of diverse molecular functions (protein-molecule interactions, transporters, transcription factors, and protein- and membrane-modifying enzymes). Most of these proteins are uncharacterized in respect to specific biological processes, while others are known to function in processes unrelated to regulating FA composition. Some of these proteins are known as components of phytohormone-mediated signaling pathways that could be involved in regulating FA composition under various environmental stresses, or their genes have been shown to be stress responsive. All these genes represent potential candidates for experimental validation and some of them will be discussed in the context of their association with relevant phytohormone-mediated signaling pathways and/or stress responsiveness.

#### 4.3.1 Abiotic stress-related candidate genes

For the OA-related FA ratios, two adjacent linkage groups (1.4 and 1.5) with three and seven SNPs (ss715580258 – 60 and ss715580261 – 315) on chromosome 1 had three SNPs significantly associated with several OA/LA-related ratios and seven SNPs above the suggestive threshold for the PA/OA ratio. Three genes (*Glyma.01g191000* - *Glyma.01g191300*) were linked with most of these SNPs and encode a tetratricopeptide repeat (TPR) domain-containing protein, KAI2-related esterase, and protein phosphatase 2C. TPR repeat-containing proteins are involved in protein-protein interactions, while protein phosphatases regulate activities of phosphorylated proteins by dephosphorylation. These types of proteins act as regulatory components of phytohormone-mediated abiotic and biotic stress responses in plants (Jung et al., 2020; Jurkiewicz and Batoko, 2018; Schapire et al., 2006; Sharma and Pandey, 2016; Zhou et al., 2021). The biological function of the KAI2-related esterase is known; this enzyme is involved in the biosynthesis of strigolactones (Liu et al., 2020), phytohormones found in root exudates promoting mycorrhizal associations and germination of parasitic plants as well as many aspects of plant development and responses to drought and other environmental stresses (Jardim-Messeder et al., 2025). Within linkage group 1.5 on chromosome 1, ss715580267 was also associated with a gene encoding a DnaJ domain-containing protein. These proteins function as molecular chaperones by maintaining protein stability under heat and other environmental stresses (Dong et al., 2025; Nagaraju et al., 2020; Zhao et al., 2022). We have identified several genes for these DnaJ domain-containing heat shock proteins that were associated with SNPs on other chromosomes. For example, *Glyma.15g146900* is associated with ss715620393 and ss715620394 and the increased PA/ALA and (PA+ OA)/(LA + ALA) ratios. This gene is a homologue of the Arabidopsis *At3g06778* gene encoding a heat-shock transcription factor involved in drought and salt stress by ABA-mediated signaling (Hwang et al., 2014). While the involvement of these DnaJ domain-containing heat-shock proteins in heat and drought stress signaling is well documented, whether they also modulate FA composition remains to be explored.

Two LA- and ALA-driven linked SNP (ss715631993 and ss715631995, linkage group 18.4) are in the coding region of the gene *Glyma.18g255000* and in the vicinity of *Glyma.18g255100*. The first gene encodes an enzyme that synthesizes inositol pyrophosphates involved in auxin, JA, and SA signaling (Wu et al., 2024), while the second gene encodes an AN1-type zinc finger protein that has been identified as a stress-associated protein (GmSAP23) (Zhang et al., 2019). An AN1-type zinc finger protein encoded by *Glyma.19g193800* also could be drought stress-related as the homologous *At2g36320* gene was identified as a drought stress memory gene in Arabidopsis (Ding et al., 2013). *Glyma.19g193800* has ss715635416 in its coding sequence and is the only stress-related candidate gene belonging to the large linkage group 19.2. The second SNP (ss715631995) was identified as part of a QTL for the soybean maturity timing (Zimmer et al., 2021). Early maturing soybean accessions show increased levels of PA and OA and lower levels of LA and ALA that could be connected to warm environments in early fall (Abdelghany et al., 2020).

#### 4.3.2 Biotic stress-related candidate genes

We identified several pathogen- and insect feeding-related candidate genes associated with SNPs predicted to modulate PA, OA, and/or ALA composition. This suggests that differences in environmental conditions in Blacksburg and Warsaw during the 2020 and 2021 growing season could have provided differential conditions conducive to plant diseases and herbivory. The SNP ss715582649 that we identified as significant for increased PA compositions and PA-related ratios was previously reported as associated with soybean resistance to *Fusarium subglutinans* (Rafi et al., 2024). This SNP is located within an intron of *Glyma.02g219700*, a homologue of the Arabidopsis gene for methyl esterase 17 responsible for converting an inactive methylated form of auxin to the active form (Yang et al., 2008; Zhang and Peer, 2017). The involvement of this gene in regulating auxin homeostasis and FA composition remains to be experimentally validated in soybean.

The *Glyma.03g229500* gene encoding a NIM1-INTERACTING 1-like isoform X2 protein is located between two other candidate lipid metabolism-related genes (*Glyma.03g229400* and *Glyma.03g229600* encoding GmG6PDH1 and a lipase/acylhydrolase, respectively, that were already discussed) for modulating FA composition. NIM1-INTERACTING proteins are known to be involved in JA and SA signaling, and plant defense against aphid feeding (Thompson and Goggin, 2006), but their involvement in altering FA composition has not been demonstrated. All three genes represent potential candidates for experimental validation of their involvement in regulating FA composition. Two genes (*Glyma.13g194500* and *Glyma.13g194600*) encoding leucine-rich repeat containing proteins were both linked to the same two SNPs (ss715615032 and 34) for the high FA/ALA-related ratios. The resulting proteins may be involved in aphid resistance by lowering the ALA availability for JA synthesis during aphid feeding (Yang et al., 2022). *Glyma.13g194500* is a homolog of the CYR-1 gene involved in resistance to yellow mosaic disease and *Phytophthora sojae* (Rahman et al., 2023; Schneider et al., 2016), while both genes may confer resistance to a root-knot nematode (Alekcevetch et al., 2021).

## 5 Conclusions

Differential environmental conditions in Blacksburg and Warsaw in 2020 and 2021 sufficiently affected FA composition in soybean seeds for mGWAS. We were able to identify several candidate SNPs and genes associated with various FA ratios in soybean oil in the context of abiotic and biotic stresses, offering new insights into potential regulatory mechanisms governing these traits. The discovery of SNPs associated with specific FA ratios and linked to genes related to various stress responses also highlights the complex interplay between the environment, phytohormone-mediated signaling, and FA composition phenotypes. The observed enrichment towards low levels of unsaturation in soybean oil as a response to a combination of several environmental stresses is desirable for developing health-promoting high-OA cultivars. However, for other practical applications and for seed germination in colder environments, high PUFA content in seed oil is preferrable. Understanding how these genes influence FA composition could have significant implications for breeding efforts aimed at improving oil quality in response to industry needs and climate change.

## Data Availability

The original contributions presented in the study are included in the article/**Supplementary Material**. Further inquiries can be directed to the corresponding author. mGWAS data was also submitted to the SoyBase database.
