# Supplementary material for: Identification of novel candidate genes for regulating oil composition in soybean seeds under environmental stresses
Source: Front Plant Sci. 2025 Apr 17;16:1572319. doi: 10.3389/fpls.2025.1572319 (PMC12044429; doi:10.3389/fpls.2025.1572319)
Supplement: Supplementary file 1 [file Presentation1.pptx]

## Slide 1
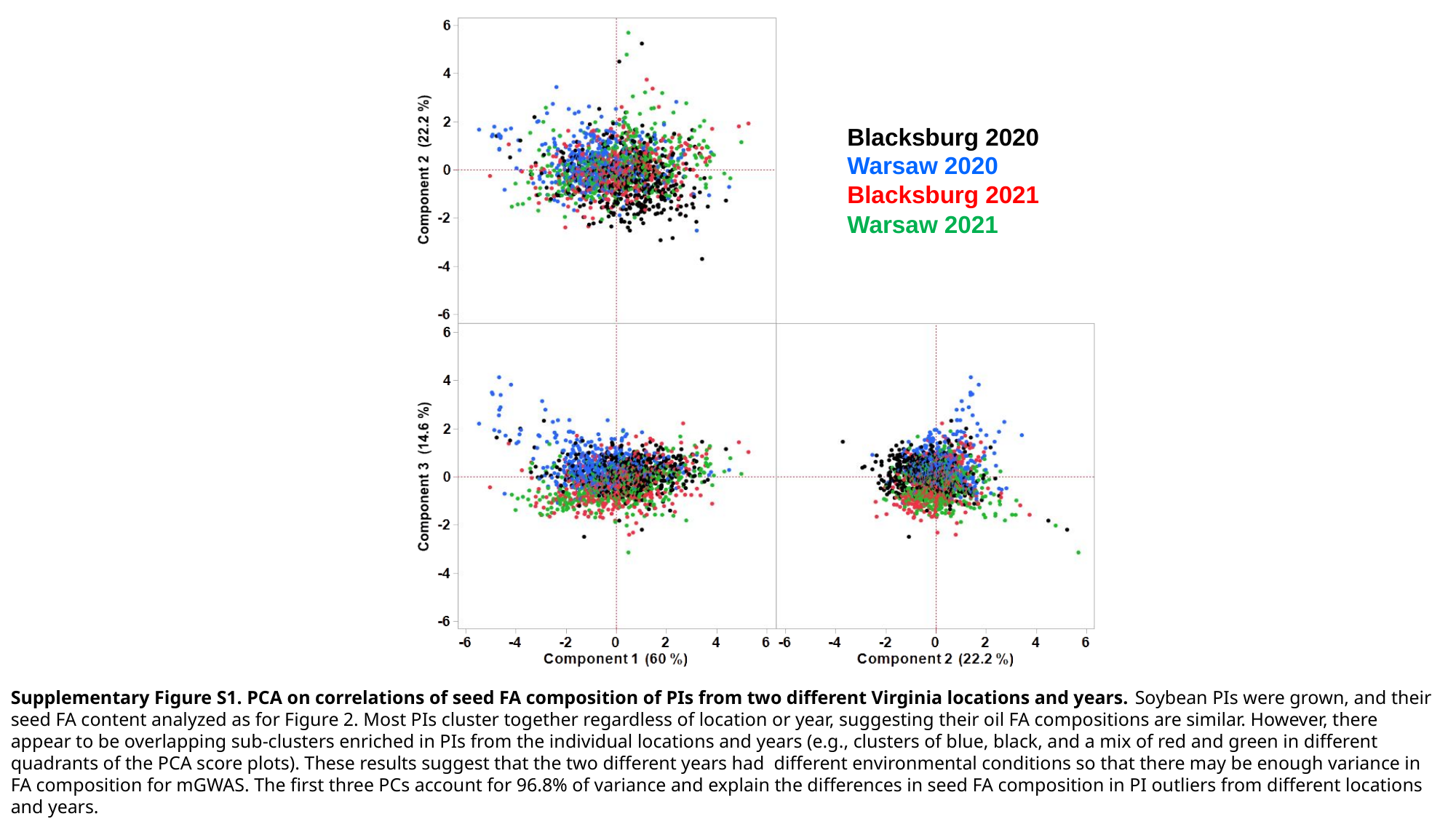

Blacksburg 2020
Warsaw 2020
Blacksburg 2021
Warsaw 2021
Supplementary Figure S1. PCA on correlations of seed FA composition of PIs from two different Virginia locations and years. Soybean PIs were grown, and their seed FA content analyzed as for Figure 2. Most PIs cluster together regardless of location or year, suggesting their oil FA compositions are similar. However, there appear to be overlapping sub-clusters enriched in PIs from the individual locations and years (e.g., clusters of blue, black, and a mix of red and green in different quadrants of the PCA score plots). These results suggest that the two different years had different environmental conditions so that there may be enough variance in FA composition for mGWAS. The first three PCs account for 96.8% of variance and explain the differences in seed FA composition in PI outliers from different locations and years.
