# Supplementary figures and images for: Identification of novel candidate genes for regulating oil composition in soybean seeds under environmental stresses
Source: Front Plant Sci. 2025 Apr 17;16:1572319. doi: 10.3389/fpls.2025.1572319 (PMC12044429; doi:10.3389/fpls.2025.1572319)

## Slide 1
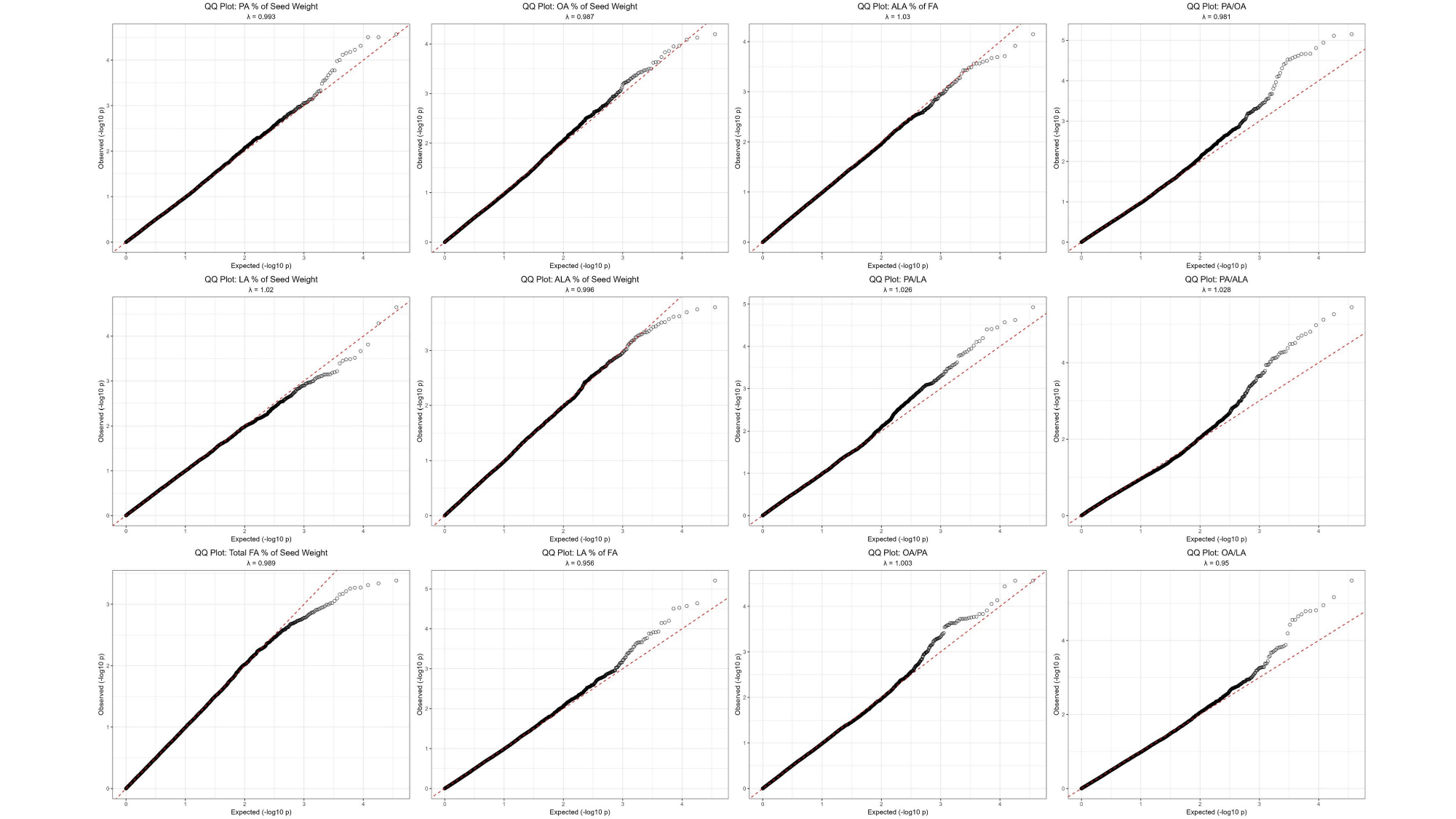

## Slide 2
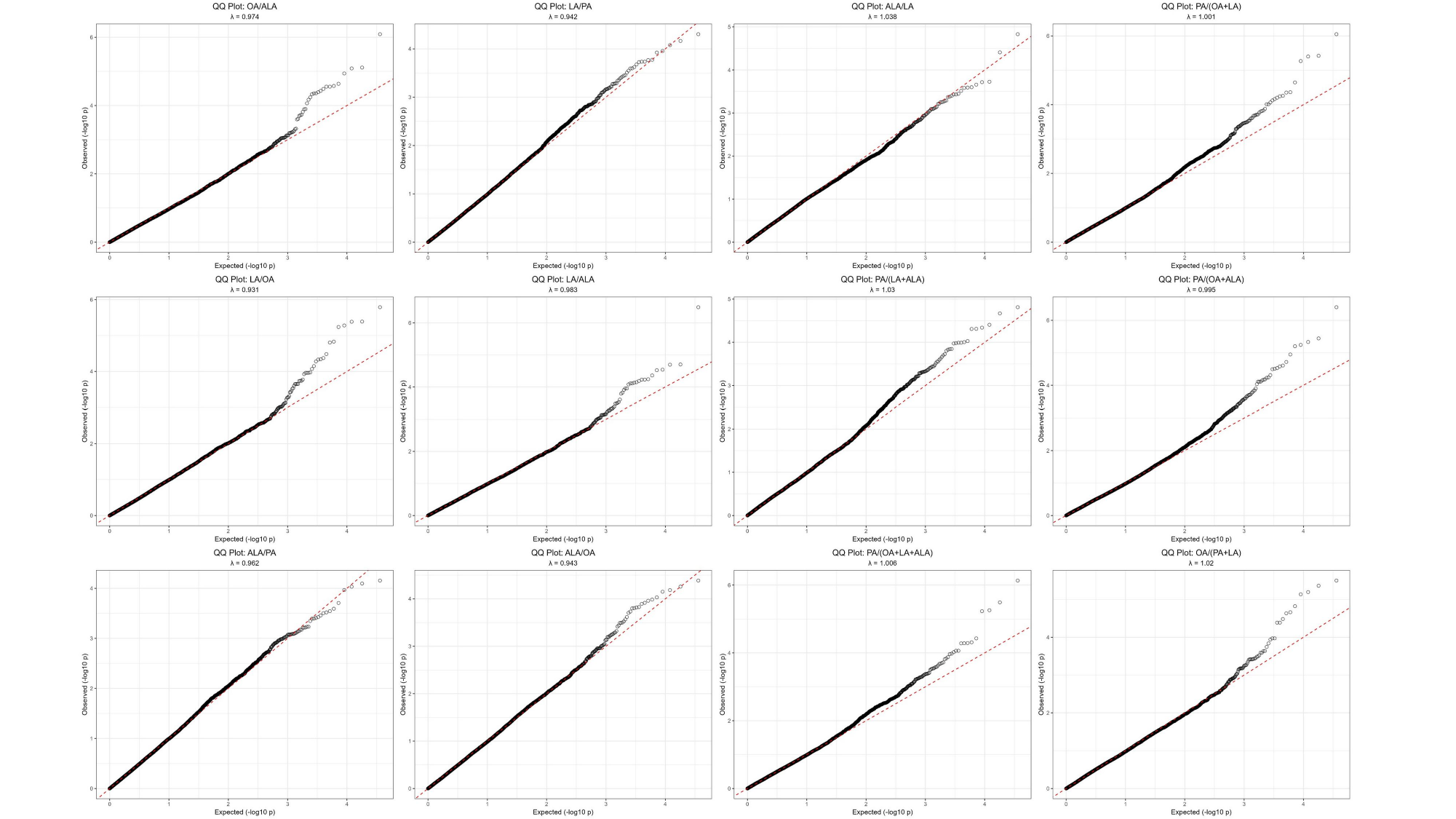

## Slide 3
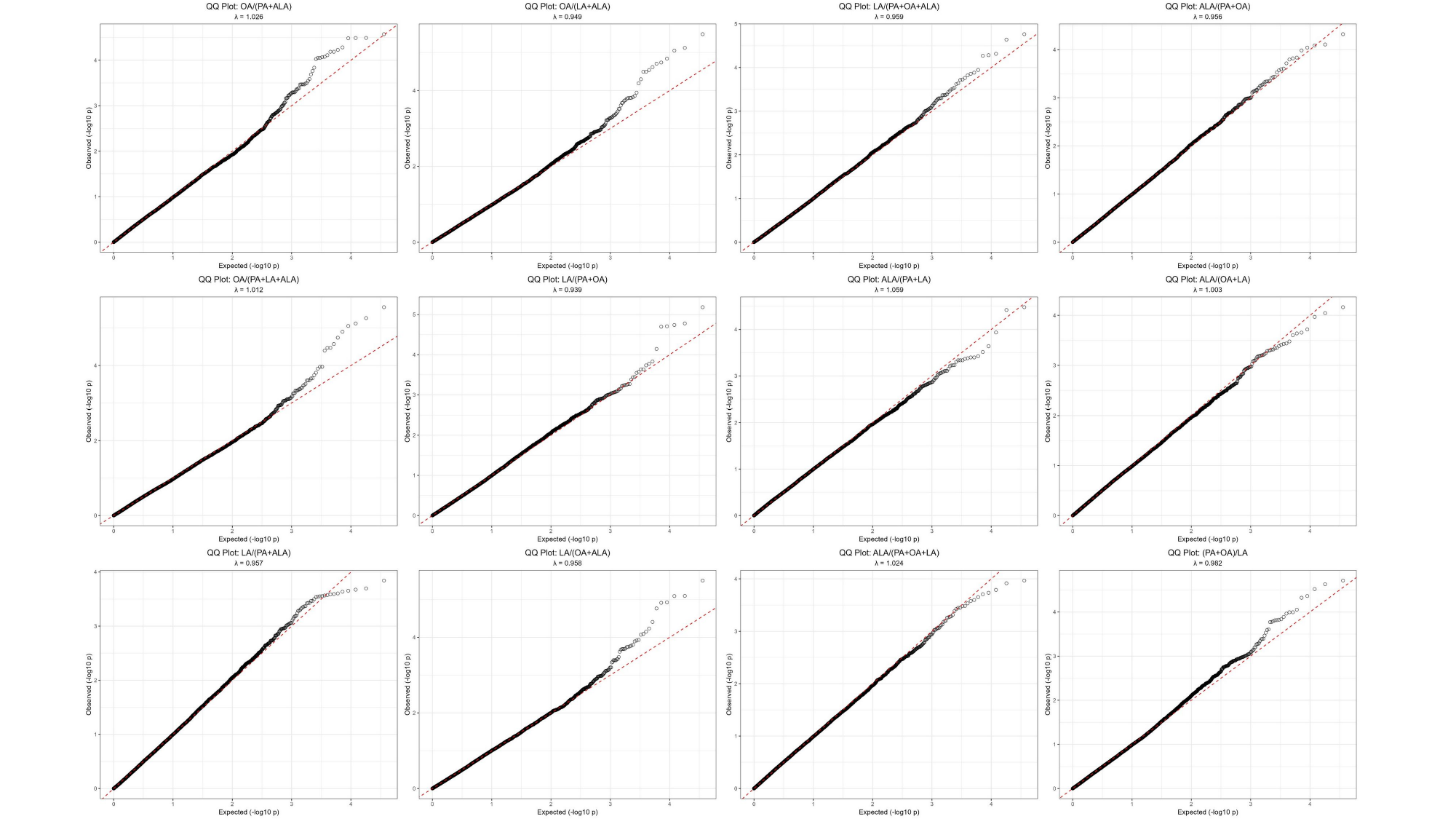

## Slide 4
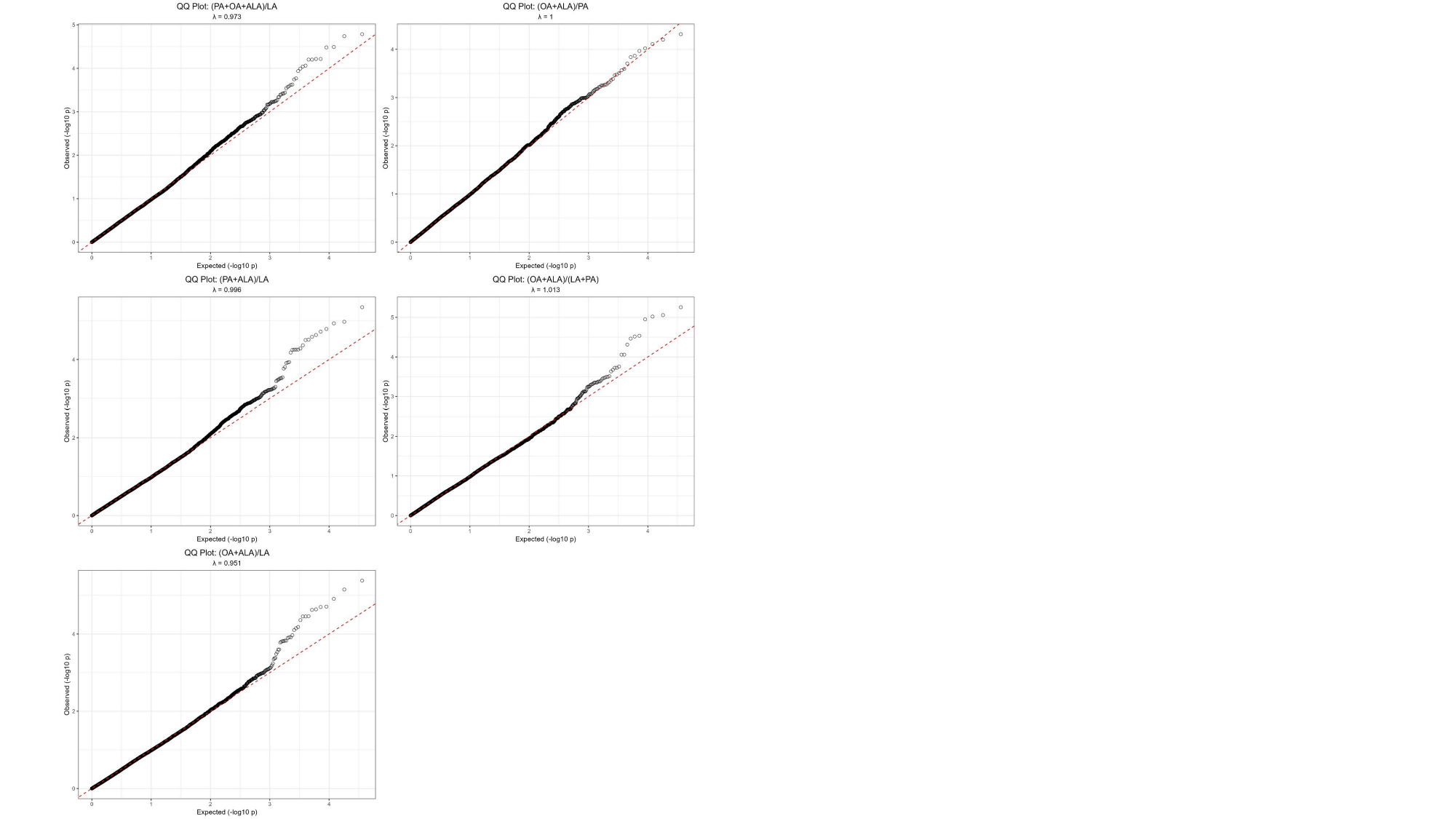

Supplement: Supplementary file 2 [file Presentation2.pptx]
