# Supplementary material for: Identification of novel candidate genes for regulating oil composition in soybean seeds under environmental stresses
Source: Front Plant Sci. 2025 Apr 17;16:1572319. doi: 10.3389/fpls.2025.1572319 (PMC12044429; doi:10.3389/fpls.2025.1572319)

## Slide 1
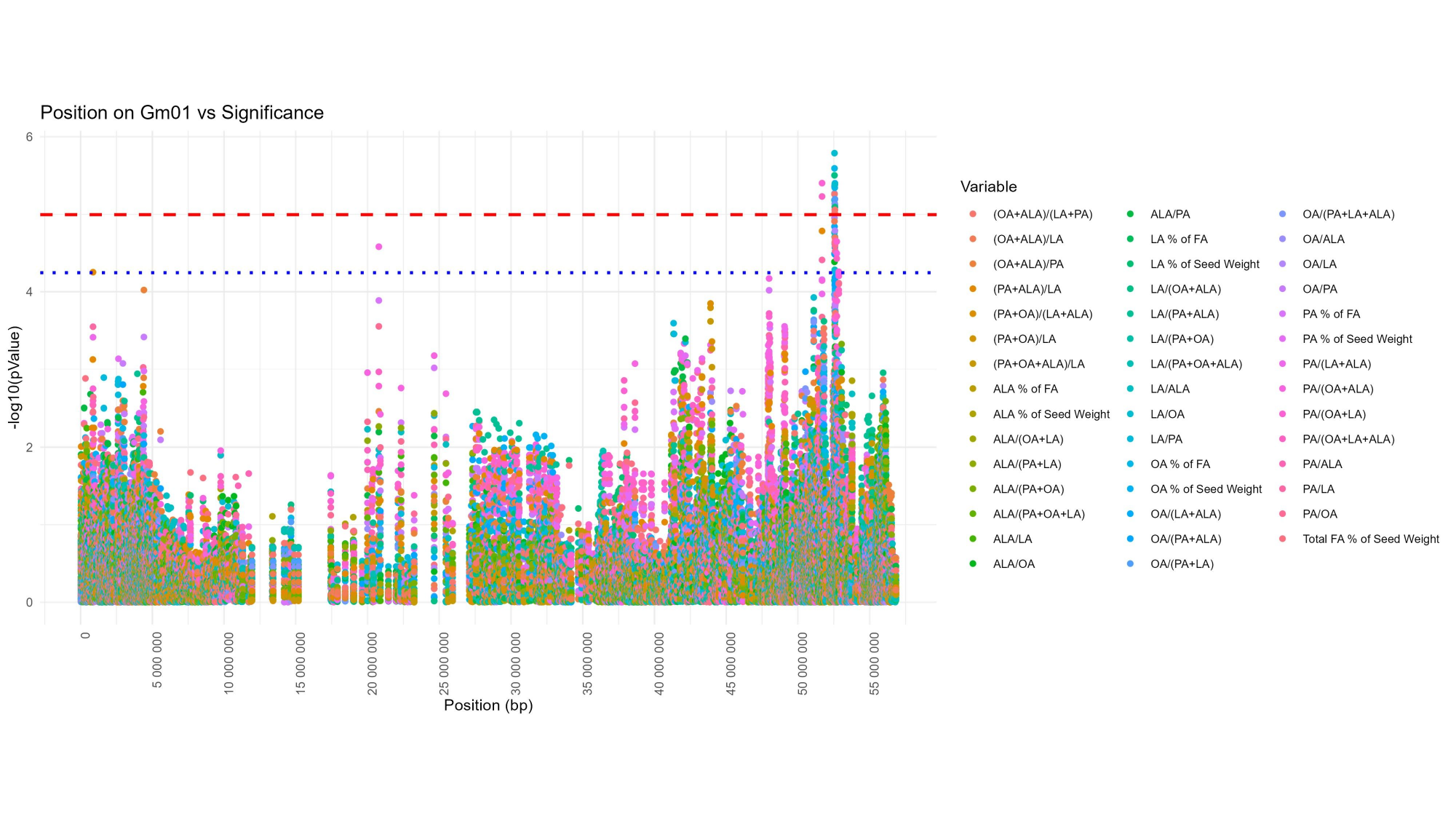

## Slide 2
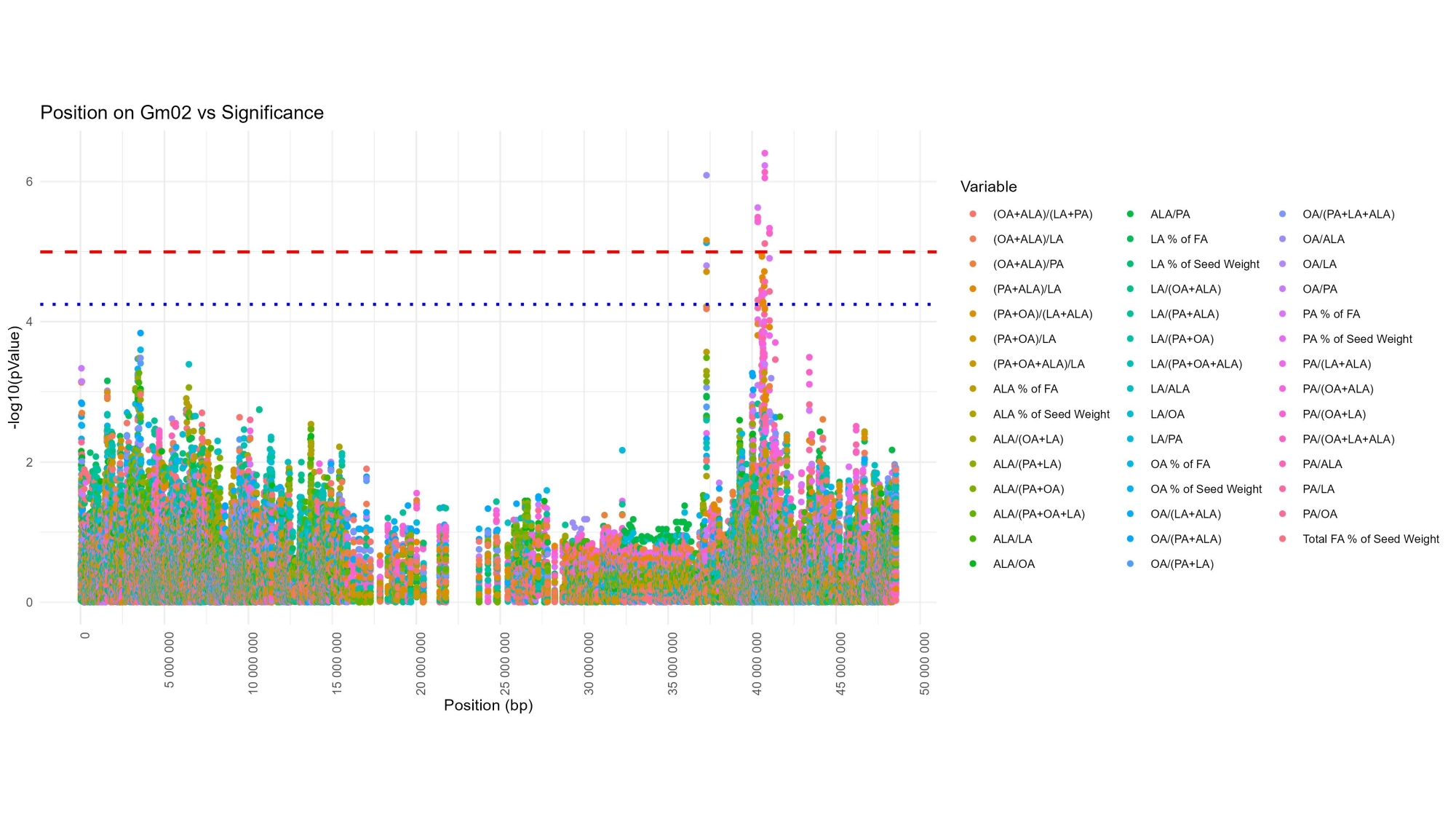

## Slide 3
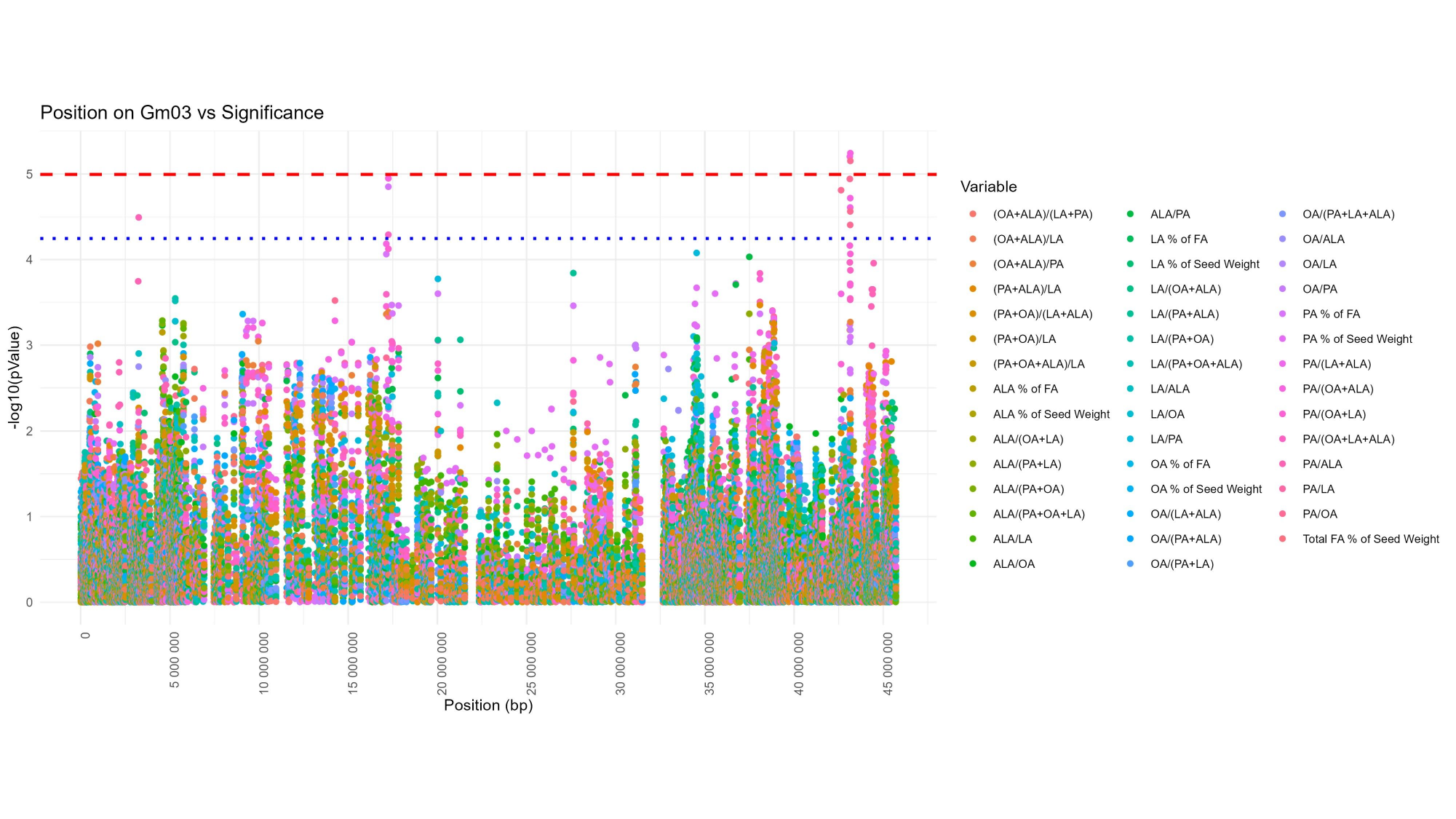

## Slide 4
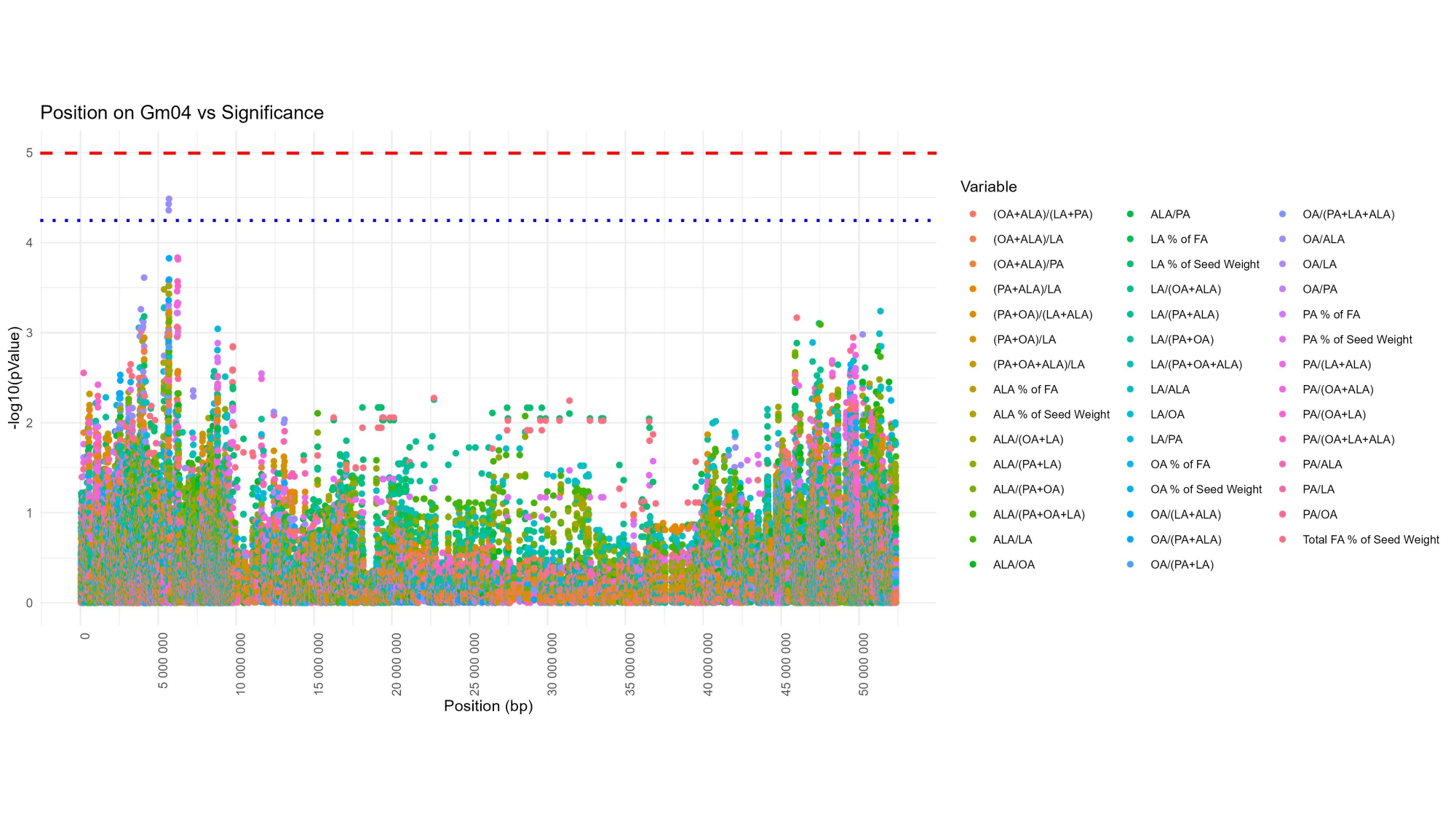

## Slide 5
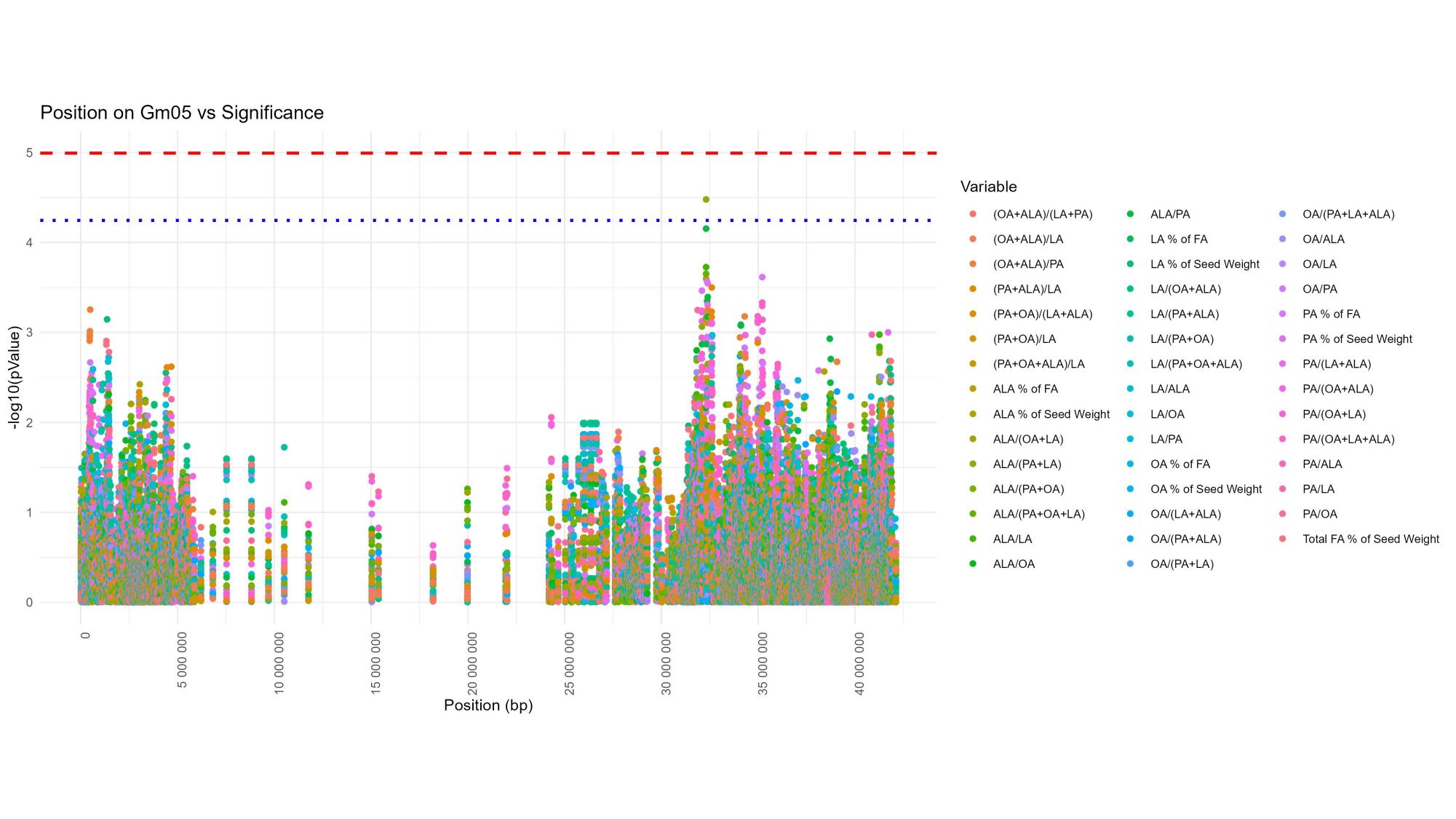

## Slide 6
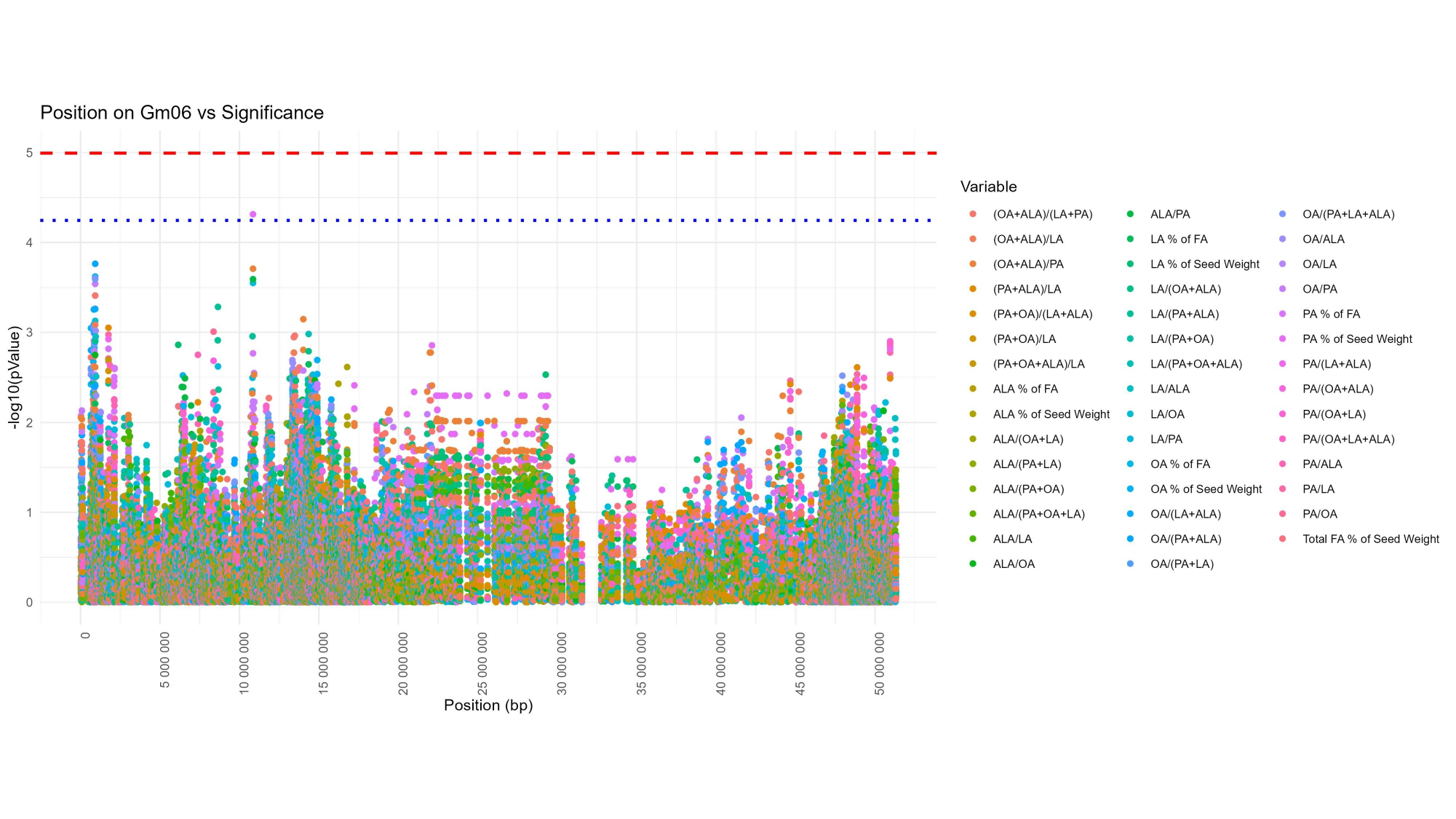

## Slide 7
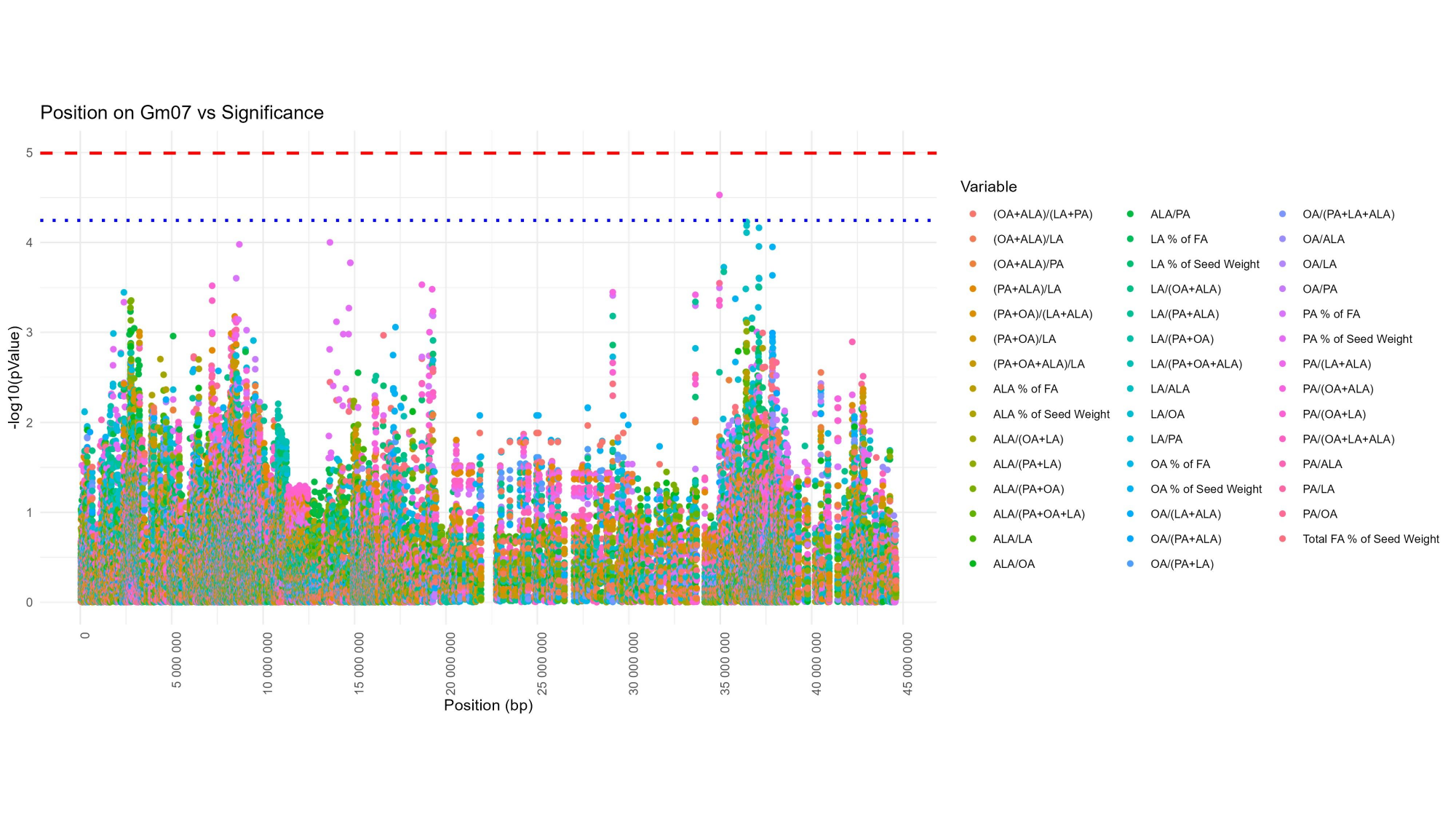

## Slide 8
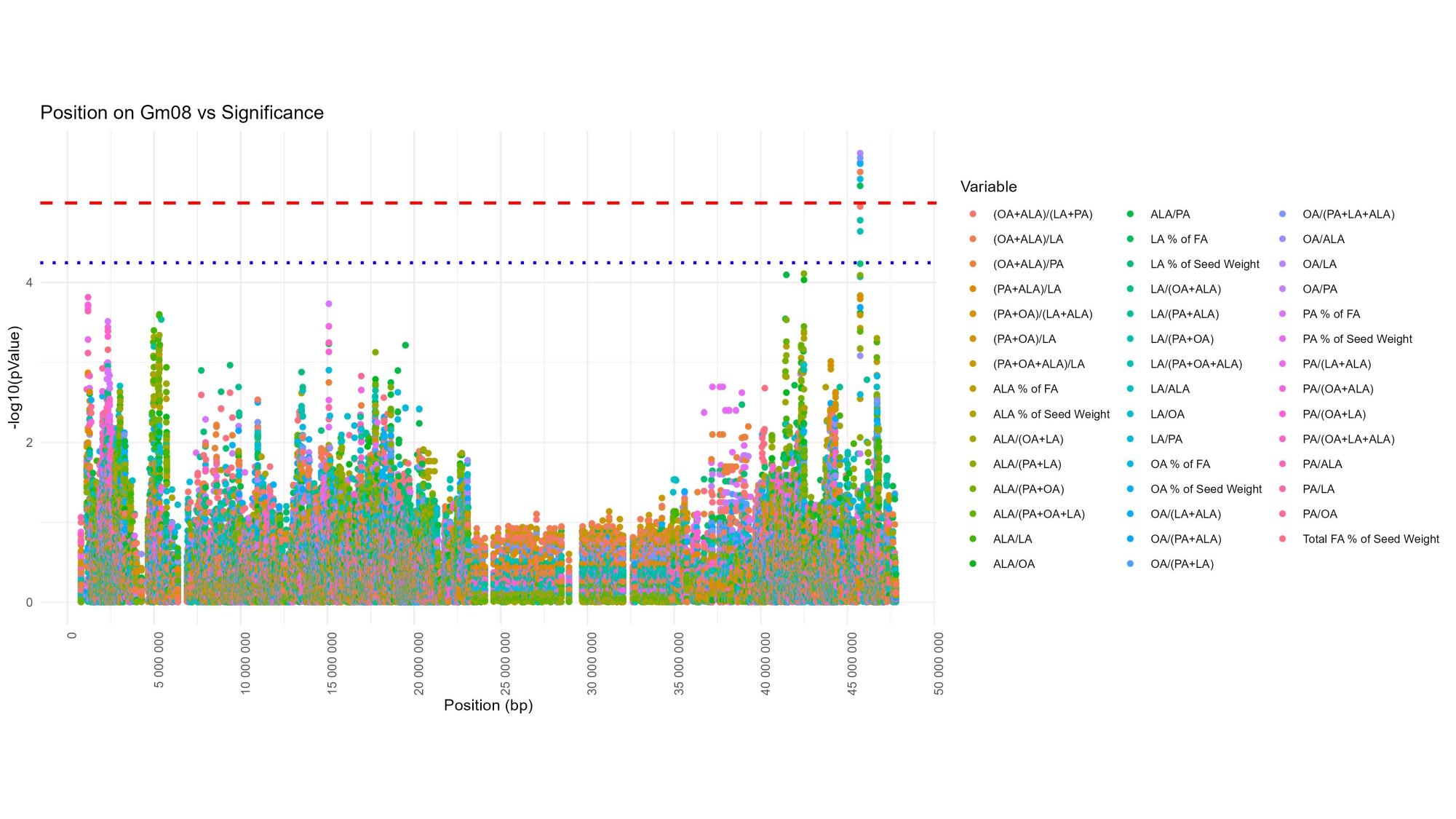

## Slide 9
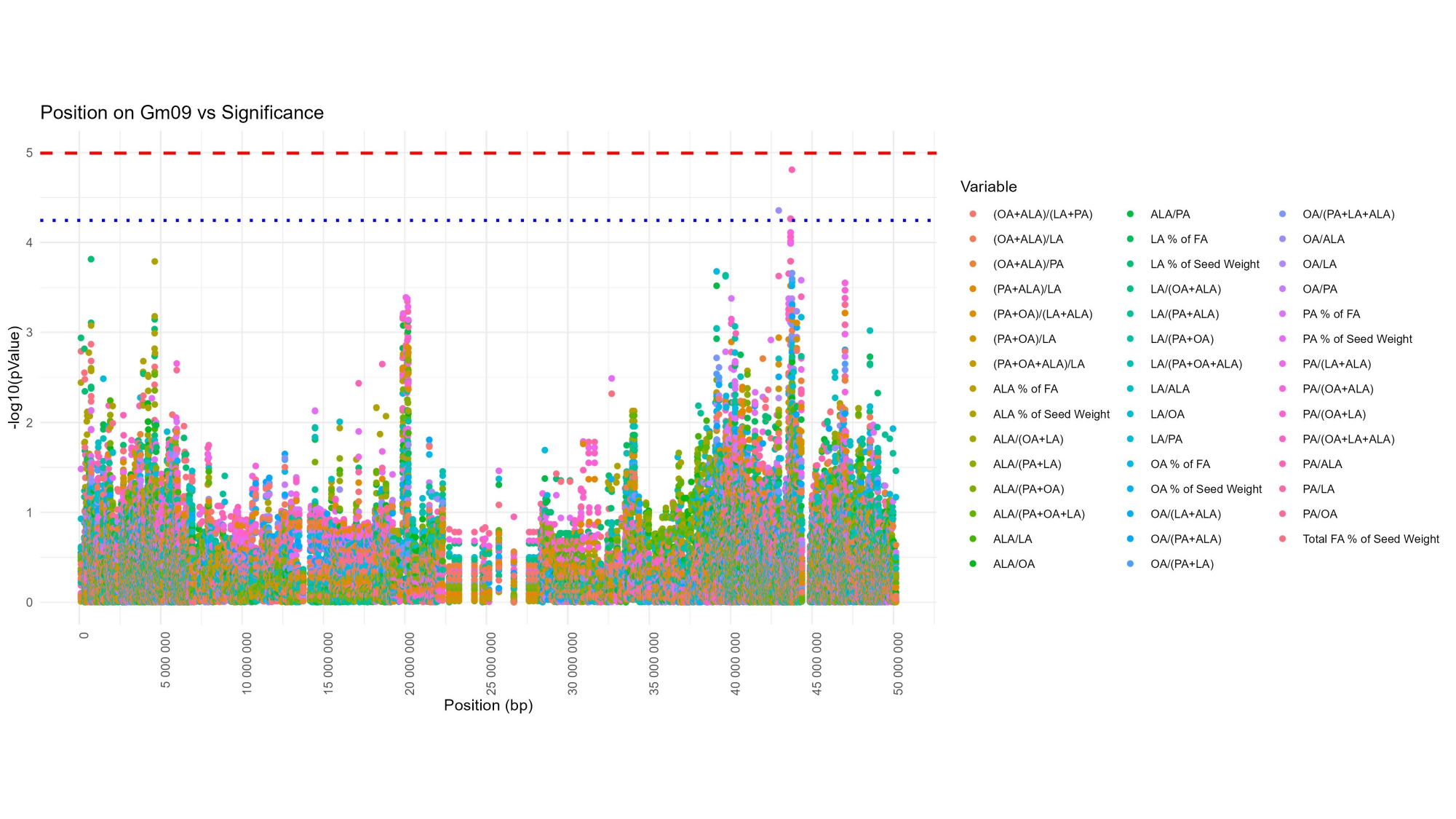

## Slide 10
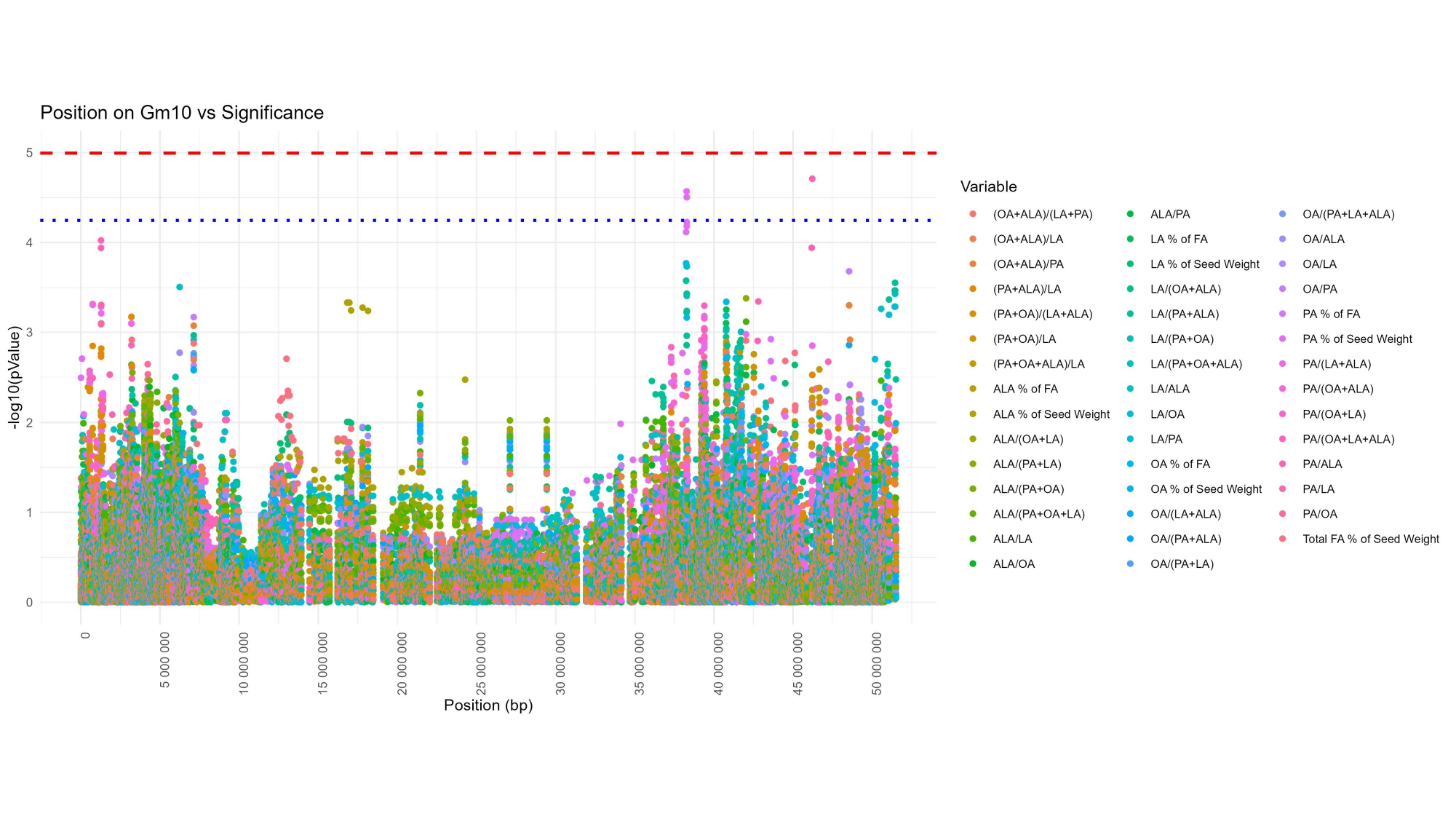

## Slide 11
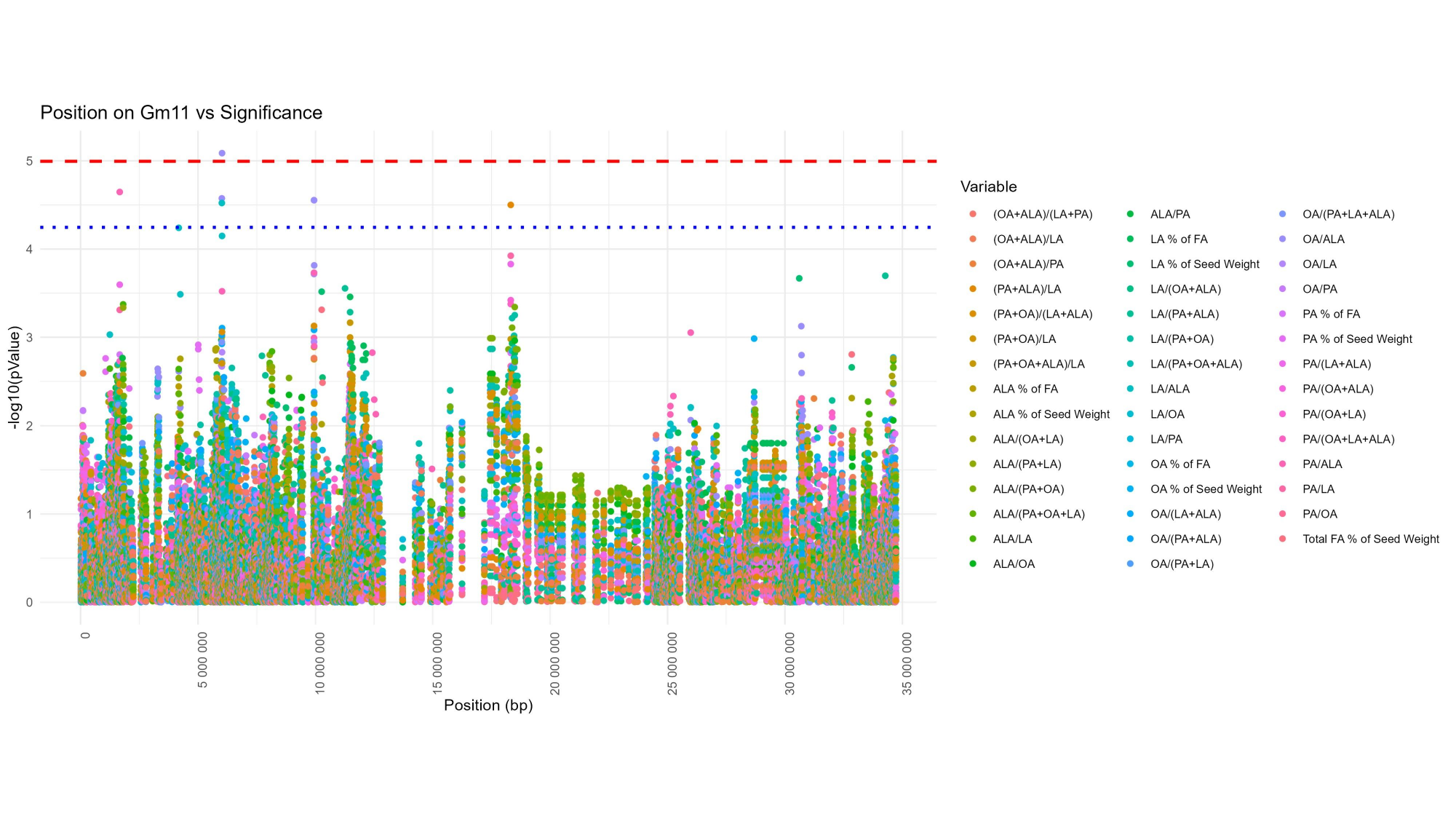

## Slide 12
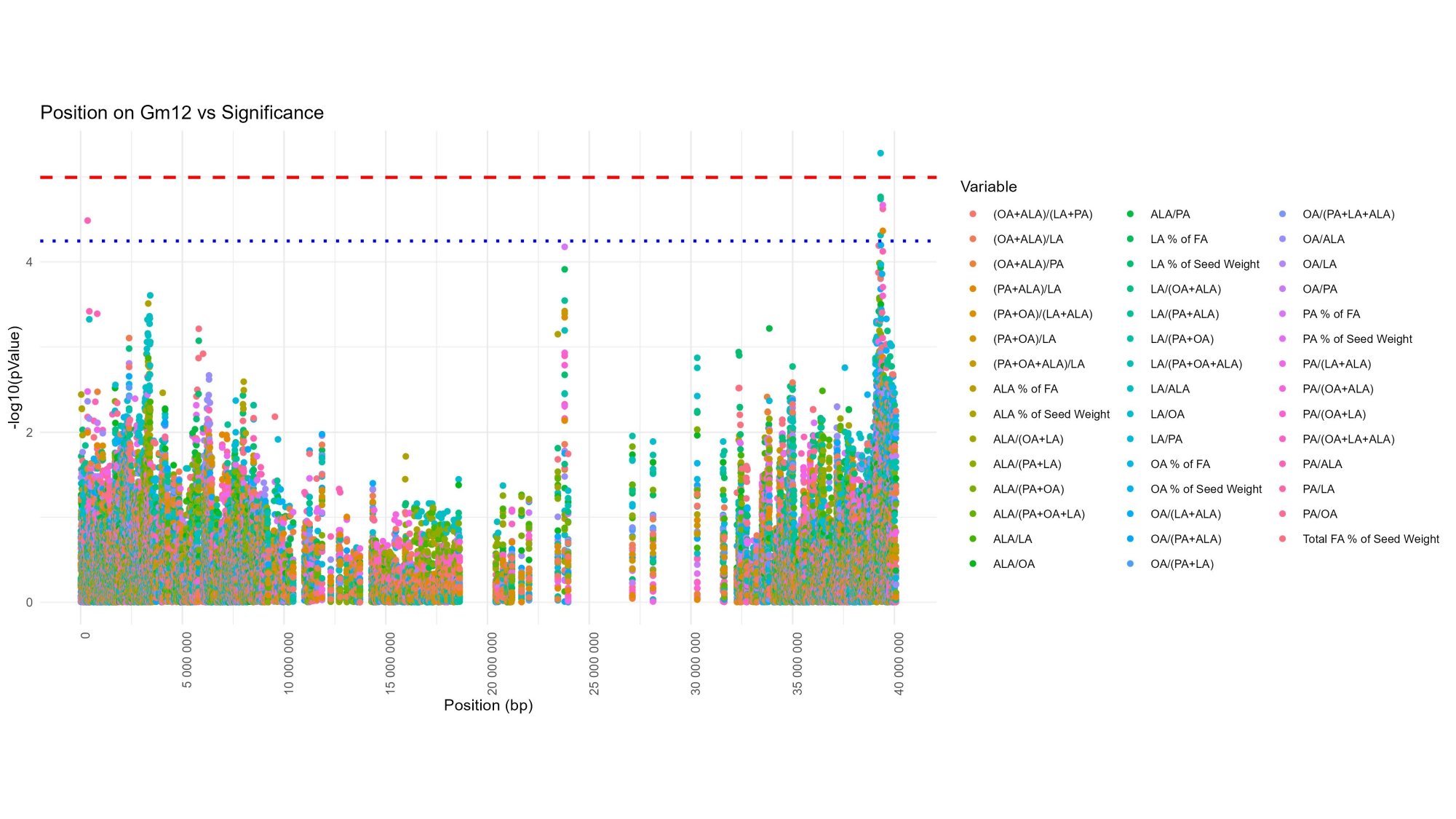

## Slide 13
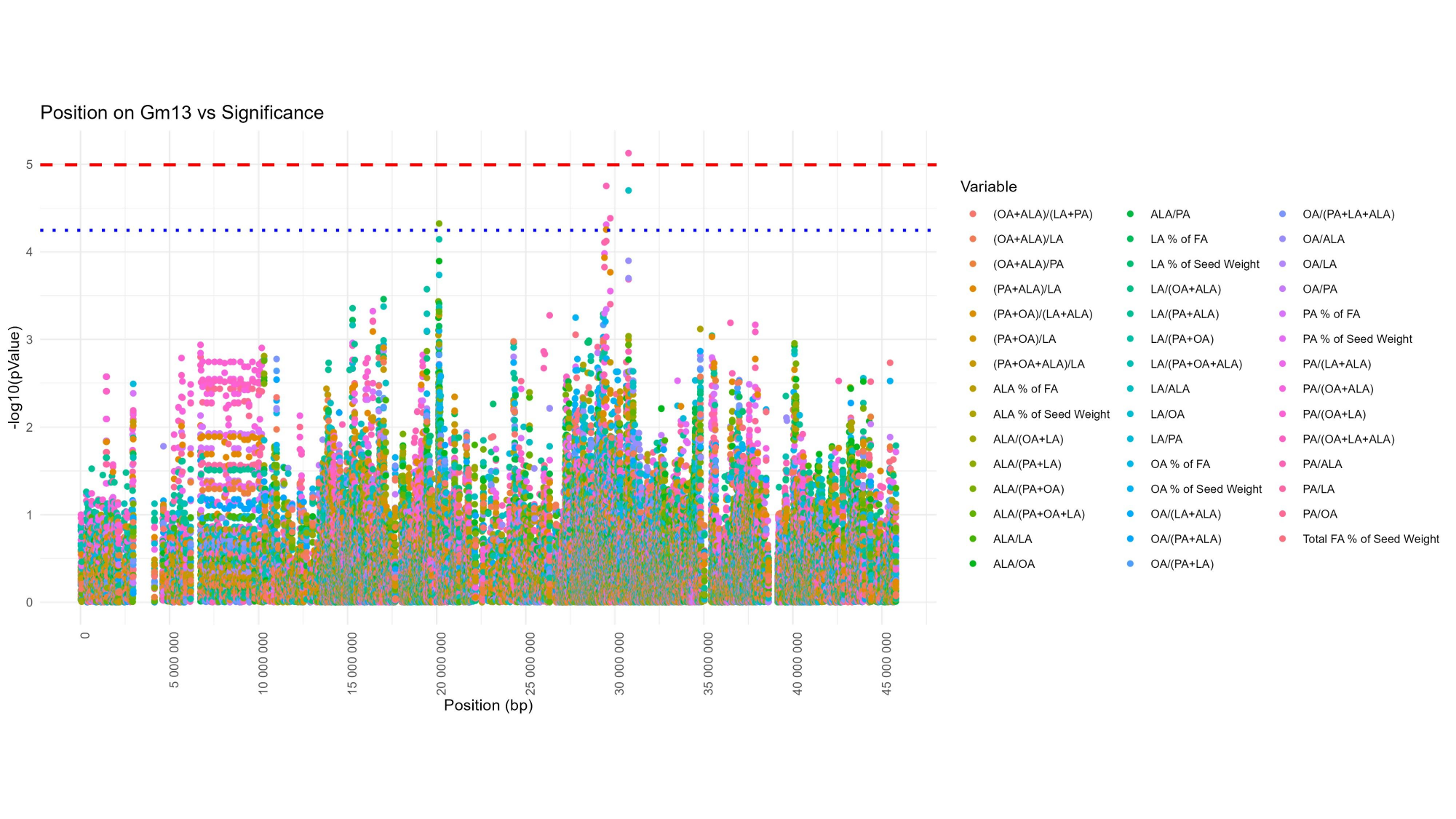

## Slide 14
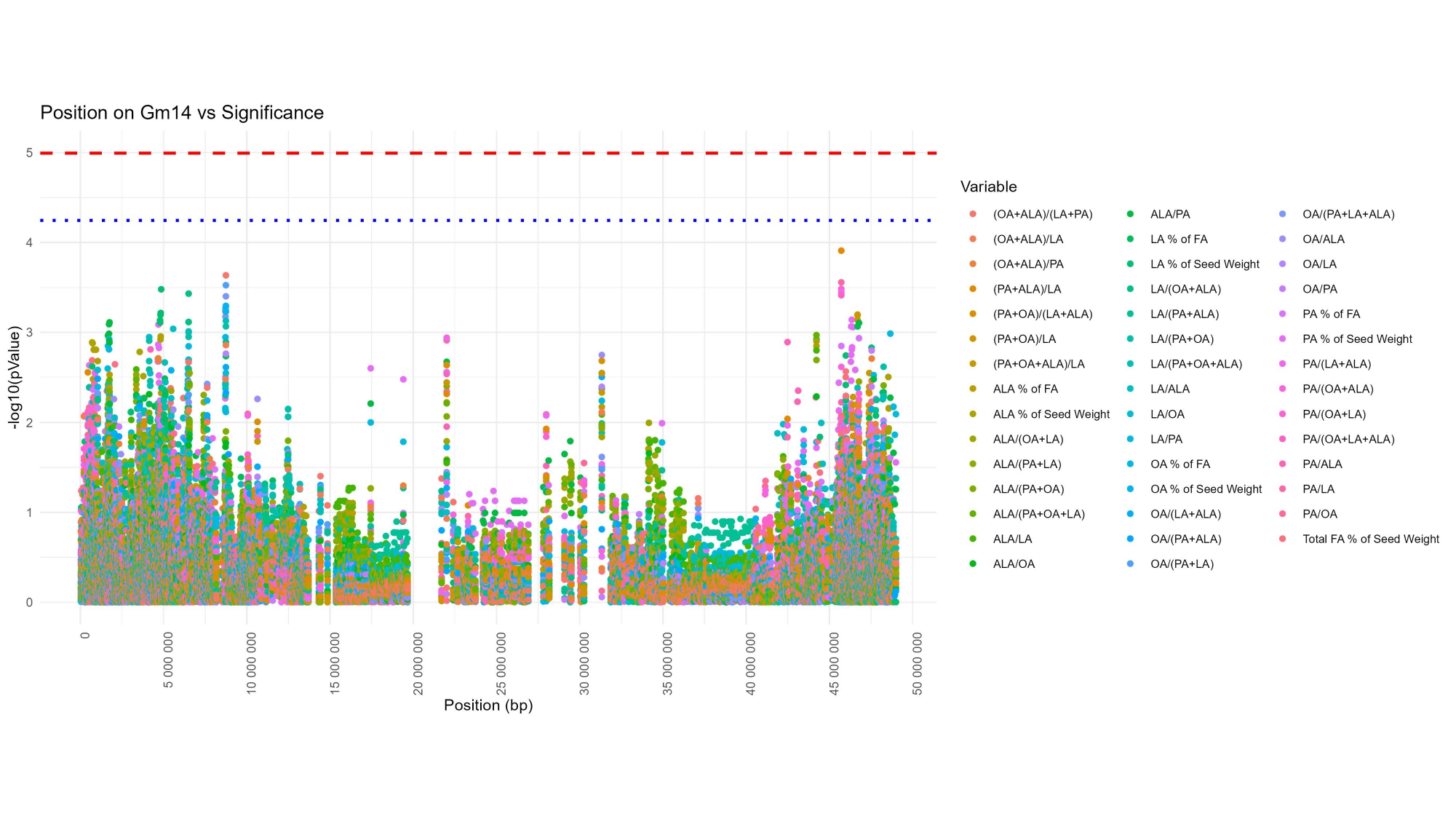

## Slide 15
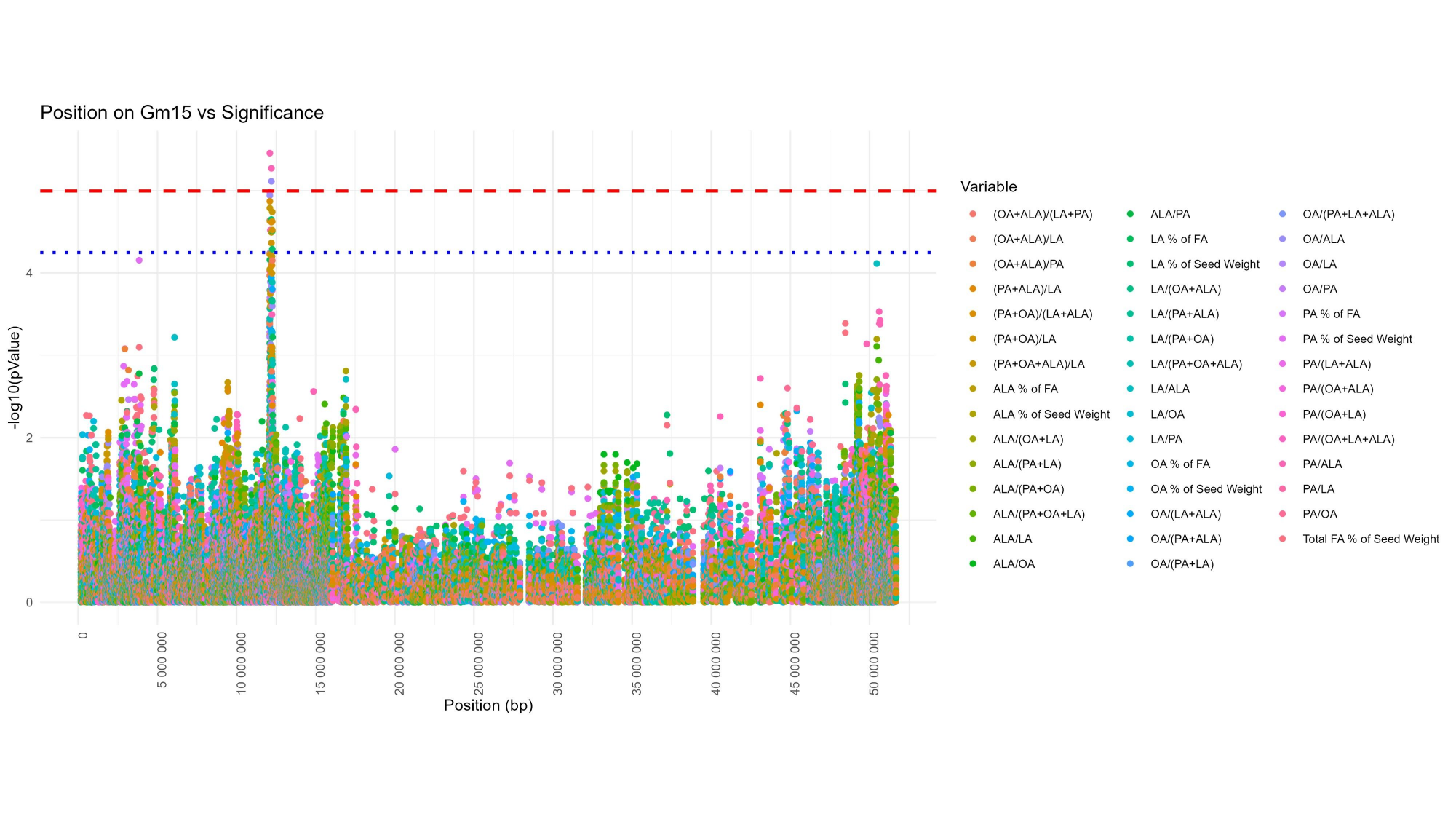

## Slide 16
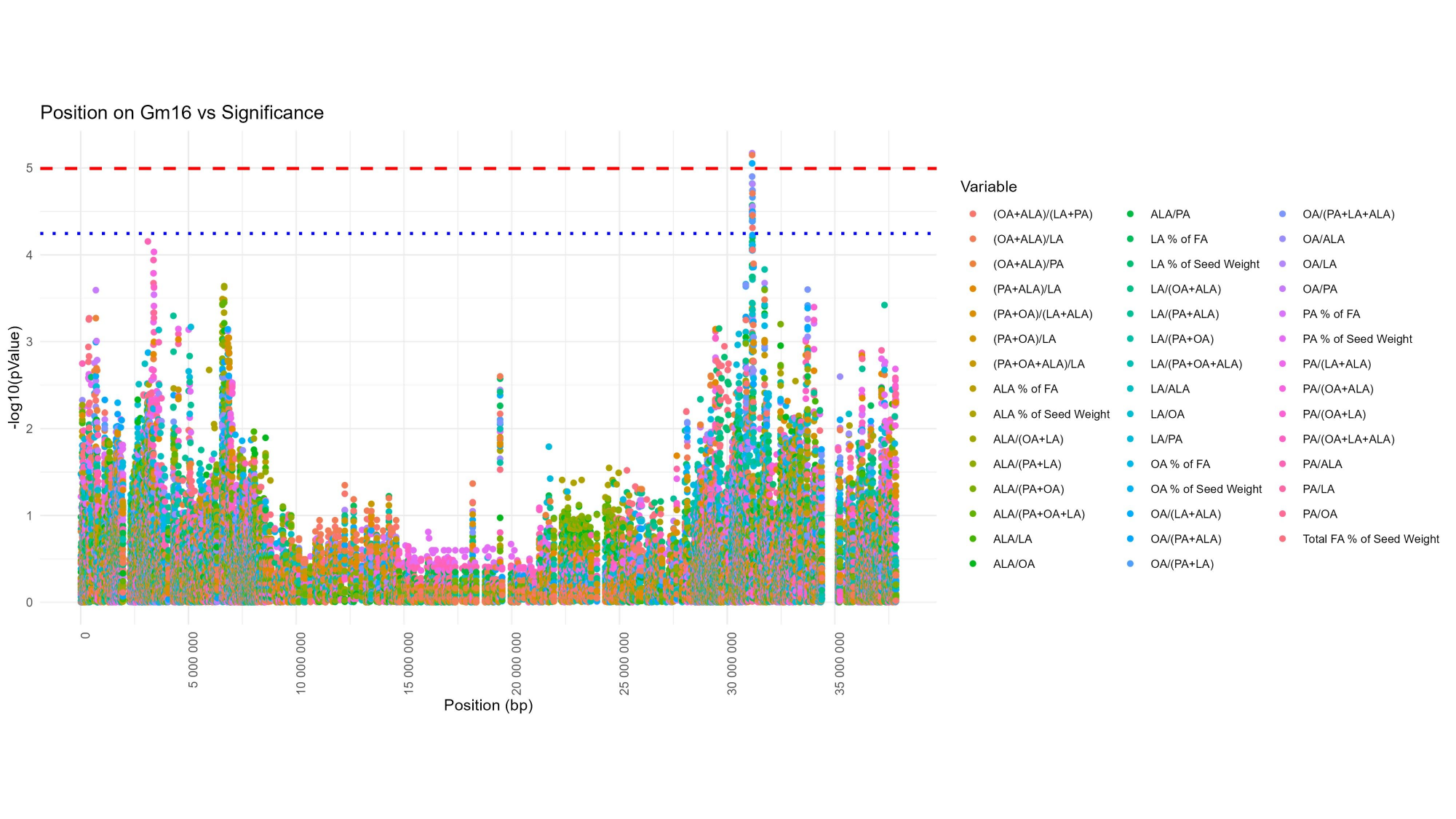

## Slide 17
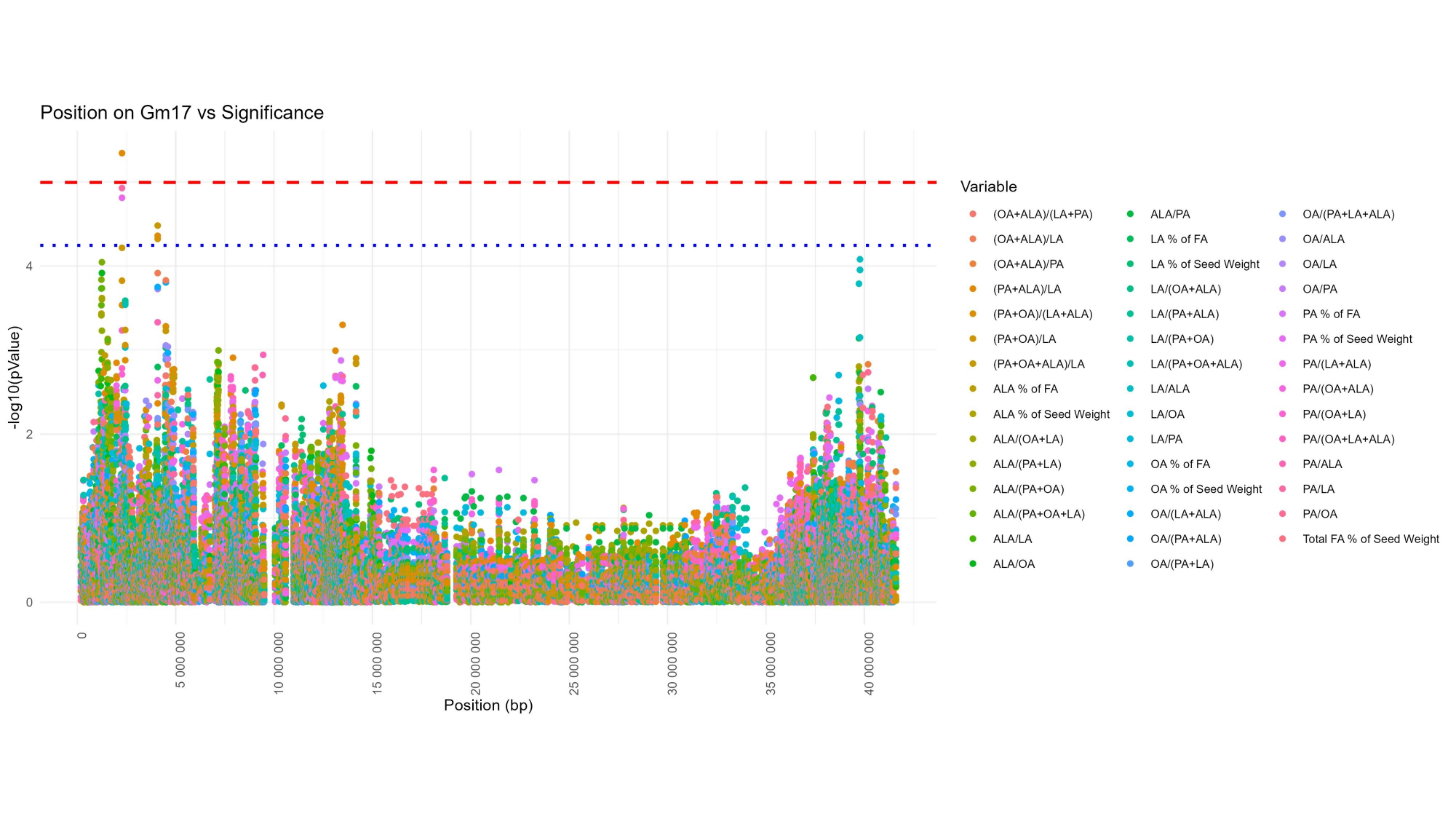

## Slide 18
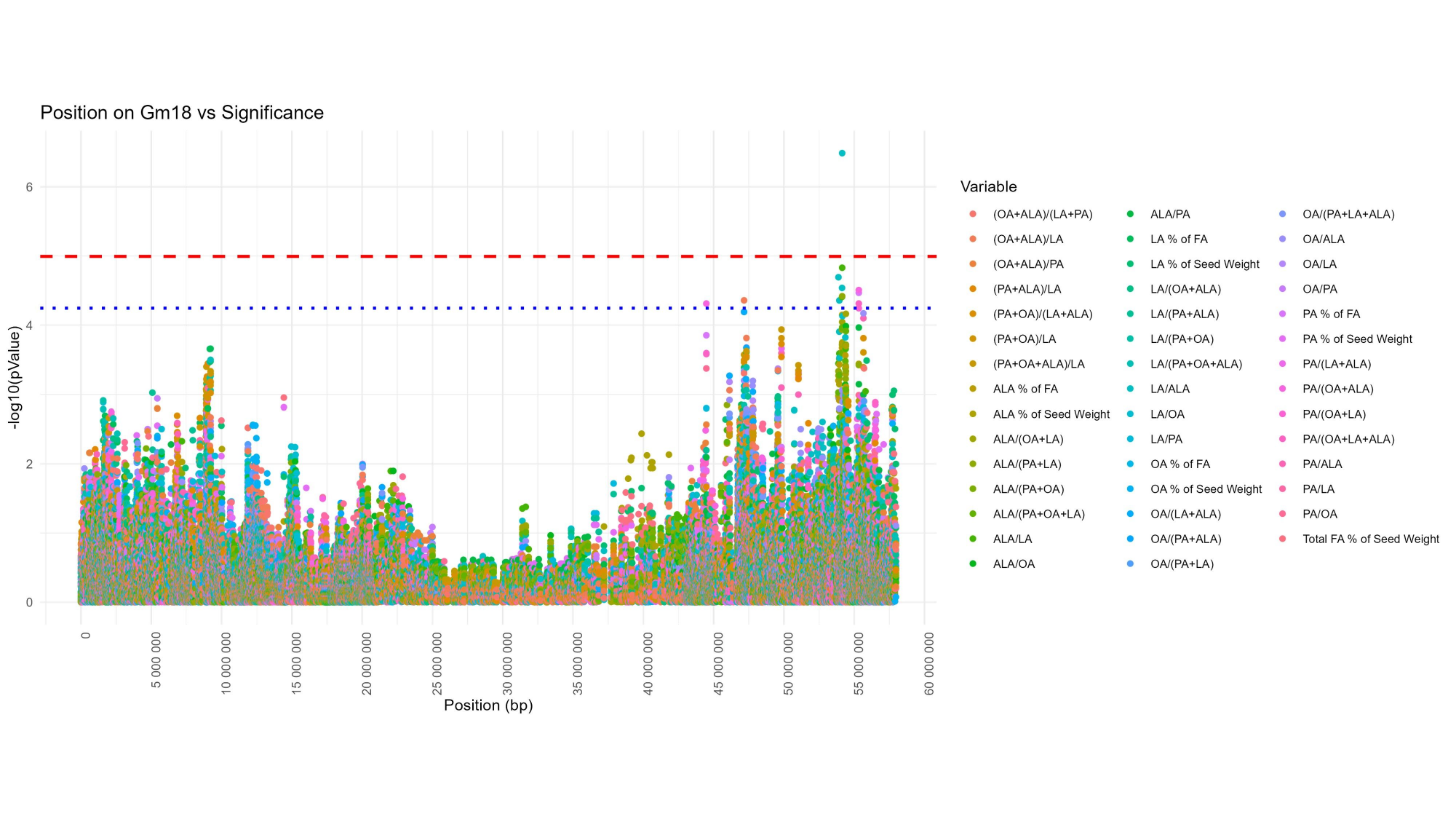

## Slide 19
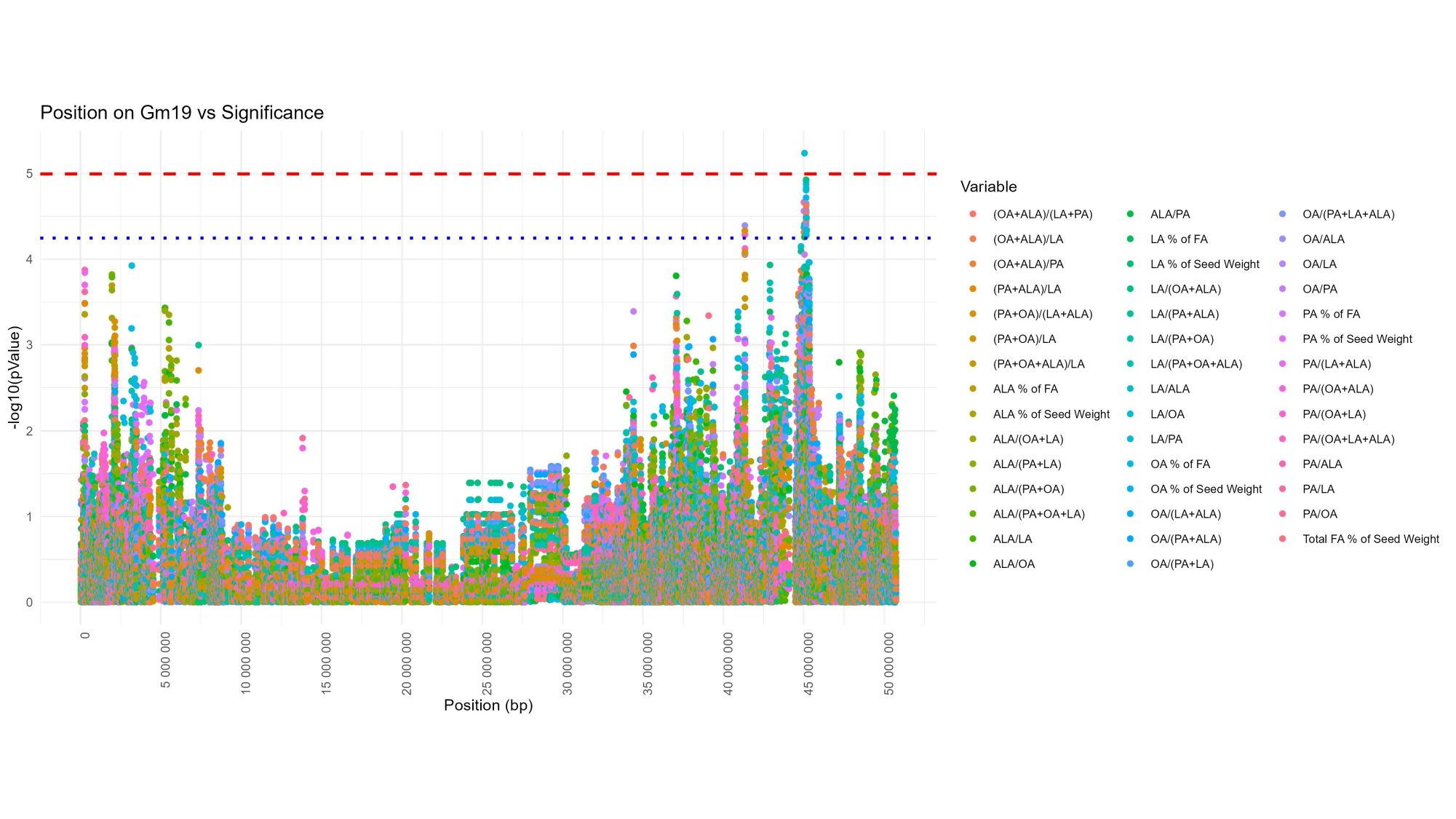

## Slide 20
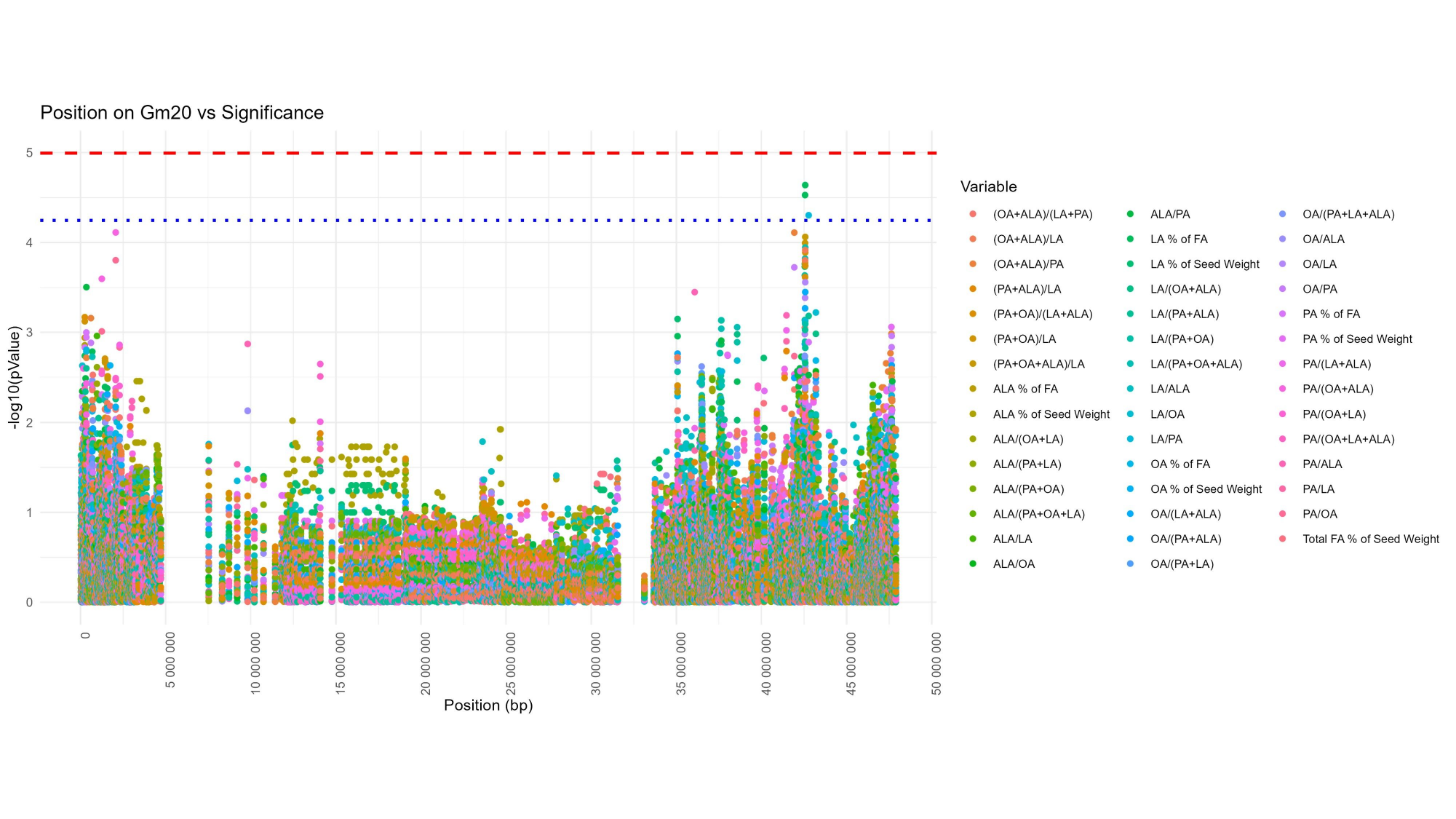

Supplement: Supplementary file 4 [file Presentation4.pptx]
